# Evaluation of the inhibitory mechanism of *Pennisetum glaucum* (pearl millet) bioactive compounds for rheumatoid arthritis: an *in vitro* and computational approach

**DOI:** 10.3389/fphar.2024.1488790

**Published:** 2024-11-21

**Authors:** Maria Sharif, Peter John, Attya Bhatti, Rehan Zafar Paracha, Abid Majeed

**Affiliations:** ^1^ Department of Biomedicine, Atta-ur-Rahman School of Applied Biosciences (ASAB), National University of Sciences and Technology (NUST), Islamabad, Pakistan; ^2^ School of Interdisciplinary Engineering and Sciences (SINES), National University of Sciences and Technology (NUST), Islamabad, Pakistan; ^3^ Crop Sciences Institute, National Agriculture Research Center (NARC), Islamabad, Pakistan

**Keywords:** rheumatoid arthritis, *Pennisetum glaucum*, network pharmacology, microarray analysis, molecular dynamic simulation, drug discovery

## Abstract

**Introduction:**

Rheumatoid arthritis (RA) is a chronic autoimmune disease characterized by synovial infiltration and pannus formation, and its rising incidence is significantly contributing to the global disability rate. Despite advances in biological drugs, no treatment has successfully cured or averted its progression. Consequently, natural drugs are being explored as alternative therapeutic strategies.

**Objective:**

This study aims to evaluate the therapeutic potential of *Pennisetum glaucum* (pearl millet) and to identify its bioactive compounds to assess their effectiveness against RA targets.

**Methods:**

The therapeutic potential of *P. glaucum* extracts was evaluated by antioxidant and anti-inflammatory assays. Gas chromatography-mass spectrometry (GC-MS) was utilized to identify the compounds in *P. glaucum* extract. The pharmacokinetics and safety profile of these compounds were studied by absorption, distribution, metabolism, excretion, and toxicity (ADMET) analysis. Network pharmacology, molecular docking, and molecular dynamic (MD) simulation were employed to identify the active compounds and their therapeutic targets in *P. glaucum* for RA treatment.

**Results:**

Acidified methanol (AM) extract of *P. glaucum* showed the highest phenolic (213 ± 0.008 mg GAE/g DW) and flavonoid content (138.1 ± 0.03 mg RE/g DW), demonstrating significant antioxidant and anti-inflammatory potential. GC-MS of AM extract identified 223 compounds. Lipinski and toxicity parameters screened out 17 compounds. Protein–protein interaction (PPI) analysis shortlisted 20 key targets in RA pathways, nine of which were upregulated in five microarray datasets. Molecular docking and MD simulations revealed that compound-7 (benzenesulfonamide, 2-nitro-N-phenyl-) and compound-9 (Pregnane-3,20-diamine, (3.beta.,5.alpha.,20S)-) bind strongly with MMP9, JAK2, PTGS2, and HIF1a compared to the reference, predicting stable interaction with these upregulated genes. Finally, PASS (prediction of activity spectra for biological active substances) analysis further validated the anti-arthritic potential of these compounds based on their chemical structure.

**Conclusion:**

This study uncovered a therapeutic drug candidate against HIF1a, MMP9, JAK2, and PTGS2 for RA from *P. glaucum* active compounds, laying the groundwork for future research.

## 1 Introduction

Rheumatoid arthritis (RA) is a persistent autoimmune condition characterized by intense pain, inflammation, synovial hyperplasia, and the infiltration of various immune cells. It poses a higher disability rate than other joint diseases and affects about 0.5%–1% of the global population (Akram et al., 2021). Notably, women are two to three times more prone to developing RA than men. According to Bayesian Age-Period-Cohort (BAPC) analysis, it is expected that by 2030, 18.23 women per 100,000 will develop RA each year, compared to only 8.34 men (Cai et al., 2023). Despite its prevalence, effective clinical treatments for RA are limited. For instance, non-steroidal anti-inflammatory drugs (NSAIDs), disease-modifying anti-rheumatic drugs (DMARDs), and glucocorticoids relieve inflammatory symptoms but take a long time to kick in, and they have severe side effects. On the other hand, biologics—advanced therapies derived from living organisms—are highly effective in controlling inflammation and disease progression by specifically targeting immune system components like cytokines (e.g., tumor necrosis factor-alpha (TNF-α) interleukin-6 (IL-6)) or immune cells (e.g., B-cells, T-cells), but they are expensive and generally target only a single molecule or pathway (Bullock et al., 2018).

**FIGURE 1:**
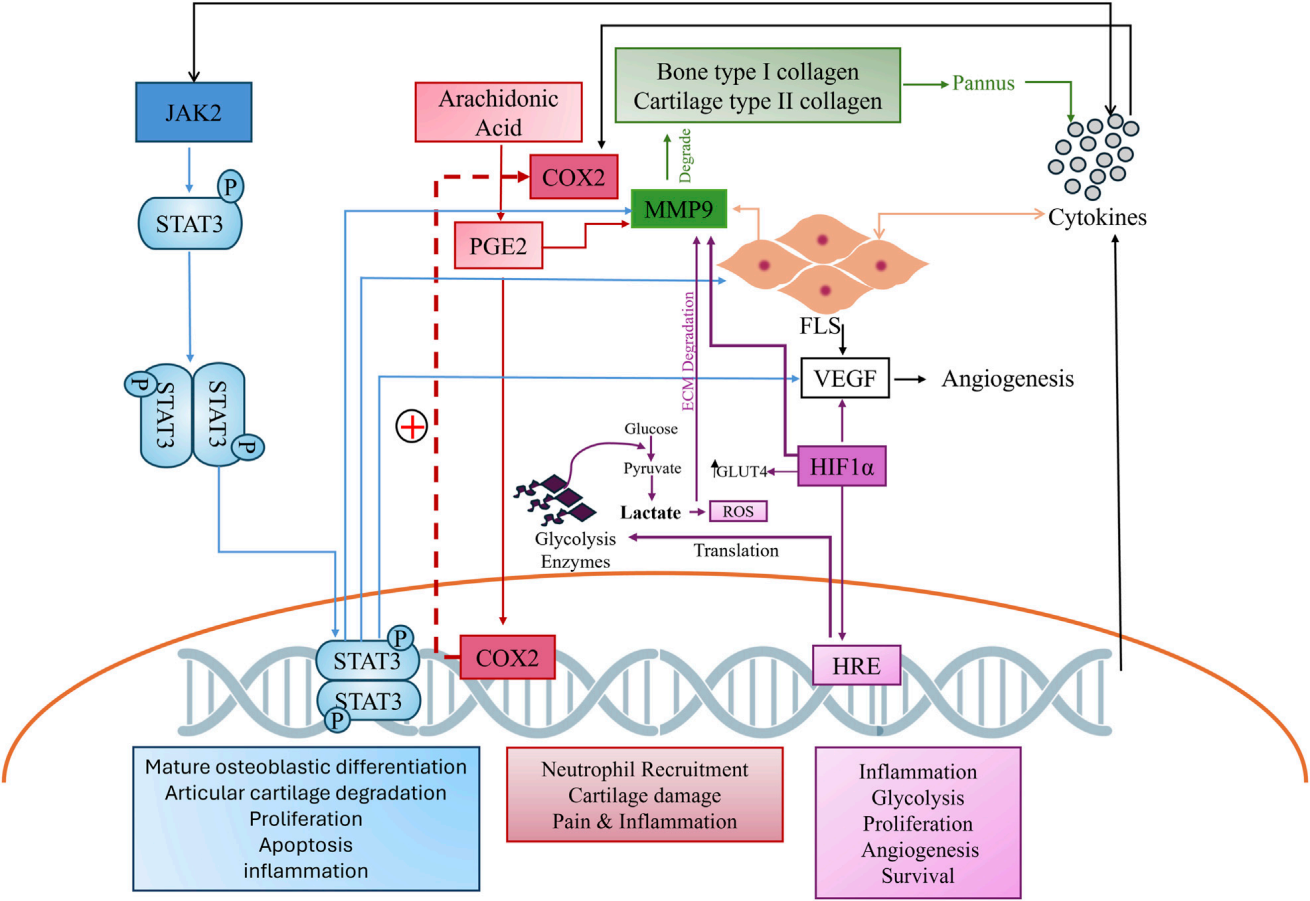
Complex cross-talk of hyper stimulated pathways in arthritis. The JAK2 pathway, shown in blue, cyclooxygenase pathway in red, MMP in green, and HIF in purple, depict the comprehensive interconnectedness of these pathways and their role in RA pathogenesis.

The complex pathogenesis of RA, which involves the continuous breakdown of bone and cartilage by matrix metalloproteases (MMPs), leads to the increased activation of proinflammatory cytokines, activating various signaling cascade like Janus kinase and signal transducer and activator of transcription (JAK/STAT) and cyclooxygenase pathways. Both pathways further stimulate proinflammatory cytokines, the rapid proliferation of fibroblast-like synoviocytes (FLS), and the activation of MMPs (Hu et al., 2022) (Ma et al., 2021). Similarly, hypoxia inducible factors (HIF) play a crucial role in exacerbating the pathogenesis of RA by stimulating the vascular endothelial growth factor (VEGF) which leads to rapid angiogenesis. A hypoxic environment also favors rapid glycolysis, resulting in the production of reactive oxygen species (ROS) which further leads to extra-cellular matrix (ECM) degradation by MMPs and inflammatory cytokines (Figure 1) (Li et al., 2023). Such complex pathogenesis requires multitargeted therapy to manage the disease. Given these constraints and the high disability rate of RA, there is a global effort to develop new drugs with fewer side effects and greater therapeutic potential. The search for novel biomarkers to alleviate RA symptoms remains a key focus.

**FIGURE 2:**
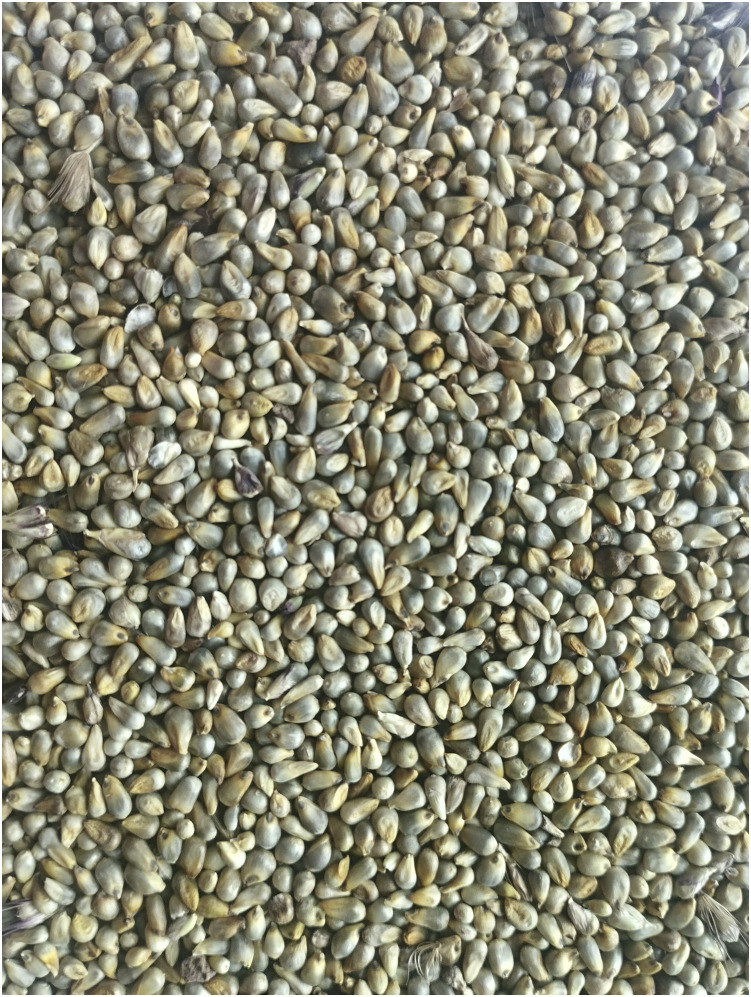
Grains of *Pennisetum glaucum*.

The safety and effectiveness of medicinal plants in enhancing human health are well-supported. Pearl millet, formally known as *Pennisetum glaucum*, is a member of the Paniceae family that is frequently consumed in tropical areas, particularly in Africa and Asia, to manage various ailments. Millet-based diets are anti-oxidant rich and have the potential to reduce inflammation in chronic alignments such as cancer and cardiovascular pathologies (Bhattacharya, 2023). Nani et al. demonstrated that *P. glaucum* consumption can significantly reduce the severity of diabetes in experimental models (Nani et al., 2015). Hegde et al. found that the inclusion of kodo millet in the diet alleviates hyperglycemia and cholesterol levels (Hegde et al., 2005). *P. glaucum* is also recommended for alleviating stomach ulcers due to its alkalizing nature. Antioxidants such as lignin and phytonutrients make them cardioprotective, and the presence of significant magnesium content helps modulate blood pressure, stress, and the respiratory symptoms of asthma. Moreover, the presence of elevated phosphorus content aids in bone growth and ATP production. It is notably safe for lactating women, the elderly, and convalescents (Pei et al., 2022). *P. glaucum* has been utilized in Moroccan ethnopharmacology for treating pain, bone fractures, and trauma, but it has not been evaluated scientifically (Ali et al., 2018). Ali et al. (2018) studied the effects of an aqueous extract of *P. glaucum* (AEPG) on the lumbar vertebrae of experimental models fed on a high-calorie diet, their findings suggesting that AEPG has the potential to be a viable and cost-effective alternative for treating bone loss and osteoporosis with no repercussions. *P. glaucum* is a natural repository of dietary antioxidants—mainly phenolics (melilotic acid, syringic acid, salicylic acid, para-hydroxyl benzoic acid, and vanilic acid) and flavonoids (tricin, 3, 4 Di-OMe luteolin, acacetin, and 4-OMe tricin) (Nambiar et al., 2012). The presence of these phytochemicals characterizes pearl millet as a nutraceutical. The bioactive constituents found in *P. glaucum* make it a promising candidate for drug discovery due to their considerable therapeutic potential. Our research focuses on exploring plant-based bioactive compounds to mitigate side effects associated with conventional RA treatments. This study aims to identify potential bioactive compounds from *P. glaucum* for RA through “computer-aided drug design” (CADD) to contribute to the development of novel and targeted therapies. These findings are intended to lay the groundwork for developing novel RA therapies.

## 2 Materials and methods

### 2.1 *In vitro* evaluation of therapeutic potential of bioactive compounds in *Pennisetum glaucum*

#### 2.1.1 Plant collection and extract preparation

Super bajra-1, an approved variety of *P. glaucum* (Munawwa et al., 2007), was obtained from the maize sorghum and millet program of the National Agriculture Research Center (NARC), Pakistan. Four extracts were prepared: ethanolic (E), hydro-alcoholic (HA), 1% AM, and aqueous (Aq). The AM extract was prepared by refluxing at 60 ℃ for 2 h, while E, HA, and Aq extracts were macerated for 24 h in a mechanical shaker at 37 ℃ and then filtered through Whatman filter paper no. 1.

#### 2.1.2 Total phenolic and total flavonoid content

Total phenolic content (TPC) was determined using Folin–Ciocalteu (FC) reagent. First, 1 mL of extract (10–90 μg/mL) was mixed with 2.5 mL of 10% (w/v) FC reagent. Then, 2 mL of Na_2_CO_3_ (7.5%) was added and incubated at room temperature for 30 min. Absorbance was measured by a UV-spectrophotometer at 765 nm against blank. The results were quantified as milligrams of gallic acid equivalent (GAE) per gram of dried weight of extract (mg GAE/g DW) (Truong et al., 2019).

Total flavonoid content (TFC) was determined using the aluminum chloride method. Extract/rutin of 1 mL (10–90 μg/mL) was mixed with 0.2 mL of 10% (w/v) AlCl_3_ solution, 0.2 mL (1M) potassium acetate, and 5.6 mL distilled water. The mixture was incubated for 15 min at room temperature. Absorbance was measured at 415 nm against blank. TFC was expressed as mg of rutin equivalent (RE) per gram of dried weight of extract (mg RE/g DW) (Truong et al., 2019).

#### 2.1.3 Antioxidant potential

Added to 1 mL of 0.1 mM DPPH solution was 2,2-diphenyl-1-picrylhydrazyl (DPPH) assay 3 mL of extract. Different concentrations of extracts were made at 10–90 μg/mL. The reaction mixture (RM) was incubated for 30 min in the dark. The absorbance was taken at 517 nm with a UV-VIS spectrophotometer. Ascorbic acid was taken as a positive control. Percentage inhibition was calculated by the given formula (15):

$$\%\text{ inhibition}=\left\{\left(\text{Absorbance of control }–\text{Absorbance of sample}\right)/\text{Absorbance of control})*100\right\}$$

To reduce the power assay, we employed the protocol of Oyaizu (1986), where 1 mL of extract was added to 2.5 mL of 0.2M PBS (pH 6.6) and 2.5 mL of 1% (w/v) potassium ferricyanide (K_3_Fe (CN) _6_) solution. The mixture was vortexed, followed by the addition of 2.5 mL of 10% (w/v) trichloroacetic acid and centrifugation at 3000rpm for 10 min. The supernatant (2.5 mL) was then mixed with 2.5 mL of deionized water and 0.5 mL of 0.1% (w/v) ferric chloride (FeCl_3_). The absorbance of the resulting solution was measured at 700 nm using a UV-VIS spectrophotometer, and the results were analyzed. Increased absorbance indicated higher reducing capability. Ascorbic acid was used as a standard (Aryal et al., 2019).

#### 2.1.4 Anti-inflammatory potential

The anti-inflammatory potential was analyzed via albumin denaturation and anti-proteinase assay (Truong et al., 2019). For albumin denaturation assay, 0.2 mL of egg albumin, 2.8 mL of PBS (pH 6.4), and 0.6 mL of extract at five different concentrations (10, 30, 50, 70, and −90 μg/mL) were used. RM was incubated for 10 min at 37 ℃ and then heated at 70 ℃ for 20 min to denature the albumin. After cooling, the absorbance was measured at 660 nm.

The proteinase assay RM contained 25uL of trypsin, 1 mL of 25 mM Tris-HCl buffer, and 1 mL of extract (10–90 μg/mL), and it was incubated for 5 min at 37 ℃. Then, 1 mL of 0.8% (w/v) casein was added and the mixture was incubated for 20 min, then 2 mL of 70% (v/v) perchloric acid was added to terminate the reaction. It was then centrifuged, and the absorbance of supernatant was determined at 280 nm against buffer as a blank. Aspirin was used as positive control for both assays. The percentage inhibition of both assays was measured by the following formula:

$$\%\text{ inhibition}=\left\{\left(\text{Absorbance of control }–\text{Absorbance of sample}\right)/\text{Absorbance of control})*100\right\}.$$

#### 2.1.5 Statistical analysis

All the analyses for *in vitro* assays were performed in triplicate, the results presented in mean ± SD. IC_-50_ values were calculated by the percentage inhibition in Microsoft Excel, and the statistical difference between the IC_-50_ values was determined via one-way ANOVA in GraphPad prism.

### 2.2 *In silico* evaluation of *Pennisetum glaucum* bioactive compounds and their potential targets for the treatment of rheumatoid arthritis

#### 2.2.1 Phytocompound library generation

Gas chromatography–mass spectroscopy (GC/MS) analysis of the AM extract of Super Bajra-1 was performed to generate a plant compound library. Samples were injected in split mode at a 1:25 ratio, using helium as the carrier gas at a flow rate of 1.00 mL/min. The oven temperature was programmed from 60 ℃ to 260 ℃, with specific hold times. The mass spectrometer operated at 70 eV, with temperatures set at 260 ℃ and 280 ℃ for the interface and ion source, respectively, and a mass scan range of 40–550 (Khan, 2017). This library of compounds was then used for further *in silico* studies.

#### 2.2.2 Drug likeness and ADME-toxicity prediction

The retrieved compounds were evaluated based on pharmacokinetics parameters using SWISSadme and ADMETlab, such as Lipinski’s rule of five, cacao permeability, human intestinal absorption (HIA), oral bioavailability (OB), molar reflectivity (MR), rotatable bonds (RB), protein-glycoprotein-substrate (Pgp-S), and toxicity parameters—hepatotoxicity (H-HT), carcinogenicity, AMES, hERG, and acute toxicity. Compounds that fulfill all these parameters were shortlisted for target prediction (Maradesha et al., 2023).

#### 2.2.3 Target prediction of disease and active compounds

Potential targets for active compounds of *P. glaucum* were retried from Super-PRED with a ≥50 probability and Swiss target prediction ≥0 probability, resulting in the shortlisting of targets with high confidence (Gallo et al., 2022).

To further explore the molecular mechanism of *P. glaucum* for RA, disease genes were retrieved from the CTD (Comparative Toxicogenomics Database), GeneCard, and OMIM (Online Mendelian Inheritance in Man) databases and literature using the keyword “rheumatoid arthritis”. Standard names were retrieved from universal protein resource by employing the selection “*Homo sapiens*” after deduplication (Zaru et al., 2023). Genes shared between RA and *P. glaucum* targets were identified using a Venn diagram and selected for further analysis.

#### 2.2.4 Gene ontology, enrichment analysis, and network construction

The Database for Annotation, Visualization, and Integrated Discovery (DAVID) performed functional annotation and enrichment analysis to determine the core mechanism and pathways of common targets (Huang et al., 2007). Gene functions were predicted at three levels: biological process (BP), molecular function (MF), and cellular component (CC). The species “*H. sapiens*” was chosen. From KEGG (Kyoto Encyclopedia of Genes and Genomes) enrichment analysis, top pathways were selected to establish a compound-target-pathways network to gain a clearer grasp of how *P. glaucum* exerts a multi-target effect against RA.

#### 2.2.5 Protein–protein interactions (PPI)

A PPI network was built from common genes using a STRING database. “*H. sapiens*” was employed as a reference organism. Genes exhibiting a confidence level >0.7 were selected and exported to Cytoscape 3.10.0 for hub gene identification by employing the degree algorithm of cytohubba (Chin et al., 2014). Twenty genes with the highest degree were selected.

#### 2.2.6 Microarray data analysis

To determine the clinical relevance of the hub genes, we obtained five microarray datasets (GSE55235, GSE12021, GSE 1919, GSE21959, and GSE89408) from the Gene Expression Omnibus database (Clough et al., 2024). R studio 4.3.1 was employed to explore the differentially expressed genes (DEGs). The cut-off criteria for DEGs was set at “adj. p Val <0.05 and |log(FC)| ≥ 1” or “|log(FC)| < - 1.0”. A volcano plot was generated by employing the “ggplot2” package of R to showcase both significant and nonsignificant genes. Lastly, upregulated hub genes were selected for further in-depth analysis.

#### 2.2.7 Molecular docking

Autodock was employed to perform molecular docking (Goodsell et al., 2021). Protein structures were retrieved from the protein data bank (PDB) and were refined via BIOVIA Discovery Studio. Kollmann-united and Gasteiger charges were assigned for the energy minimization of protein and ligand. Literature-reported residues were used for binding-site prediction and to position of the grid box (Supplementary Table S1). A generic algorithm with 20 runs was employed. The first binding poses with zero RMSD of atomic positions were considered highly valid, indicating strong binding affinity. BIOVIA Discovery Studio Visualizer 2021 was utilized for visualization.

#### 2.2.8 Molecular dynamic simulation

For the MD simulation, docked complexes with higher binding affinity than the reference drugs were selected. GROMACS-2020.1 software was employed for protein–ligand MD simulation. A CHARMM36 force field was utilized. CGenFF server (accessed in February 2024) was utilized to construct the ligand topology (Vanommeslaeghe and MacKerell, 2012). In total, 24 MD simulations were executed, with only those complexes that showed stability in a 30 ns timeframe being extended to 100ns. Trajectory analyses of the hydrogen-bond (H-bond), solvent-accessible surface area (SASA), radius of gyration (RoG), root-mean-square fluctuation (RMSF), and root-mean-square deviation (RMSD) were conducted by XMGRACE.

#### 2.2.9 Prediction of biological activity

The biological properties of active compounds were analyzed using PASS (Prediction of Activity Spectra for biological active Substances) (Poroikov et al., 2003) with a threshold where pharmacological activity (Pa) exceeded pharmacological inactivity (Pi). Biological activities related to RA were identified from the comprehensive array of attributes.

## 3 Results

### 3.1 *In vitro* evaluation of the therapeutic potential of *Pennisetum glaucum* extracts

#### 3.1.1 Total phenolic and flavonoid content

**FIGURE 3:**
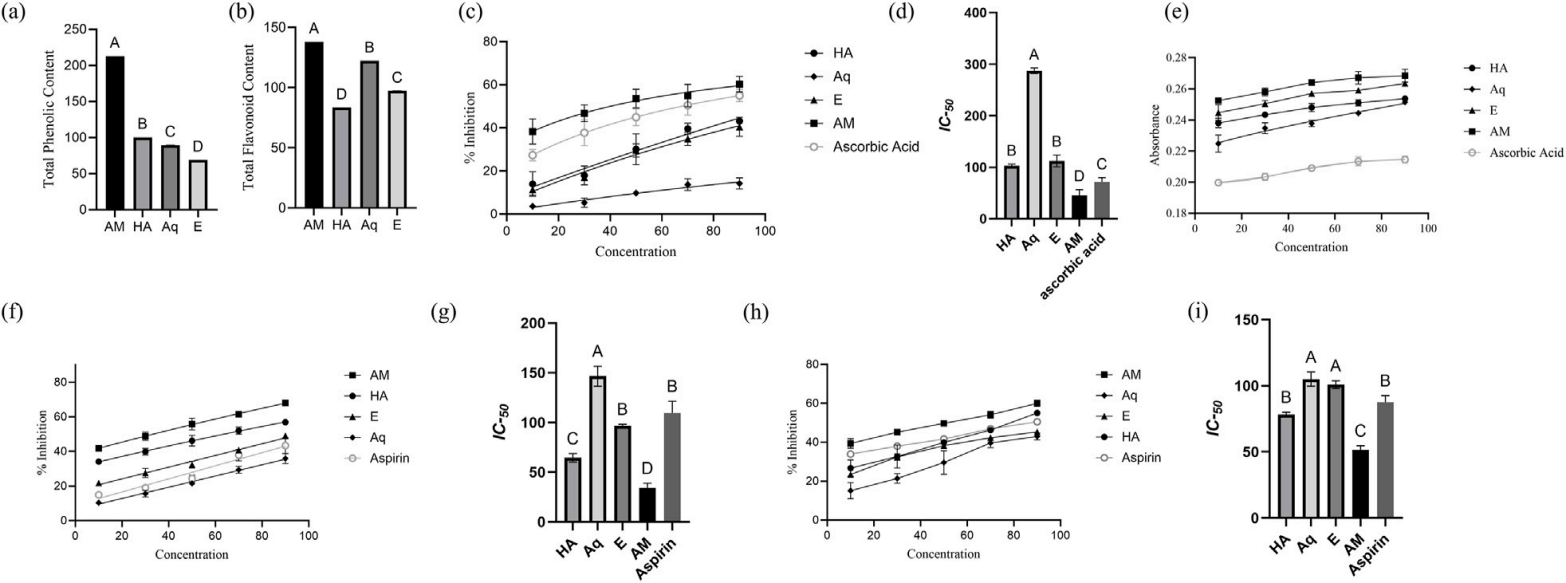
Characterization of *Pennisetum glaucum* extract. **(A)** TPC, **(B)** TFC, **(C)** percentage Inhibition of DPPH assay, **(D)** IC_50_ of extracts for DPPH assay, **(E)** absorbance of reducing power assay, **(F)** percentage inhibition of albumin denaturation assay, **(G)** IC_50_ of extracts for albumin denaturation assay, **(H)** percentage Inhibition of proteinase assay, and **(I)** IC_50_ of extracts for proteinase assay. IC_50_ were analyzed by one-way ANOVA, with different letters representing the statistical difference between different extracts. All experiments were conducted in triplicates and reported in mean ± SD.

The quantitative assessment of TPC and TFC was accessed by employing linear equations derived from the calibration curves of gallic acid (y = 0.0003x + 0.0904, R^2^ = 0.9906) for TPC and rutin (y = 0.008x + 0.005, R^2^ = 0.9996) for TFC. The results showed a significant amount of phenolics and flavonoids in all extracts. The TPC in different extracts were ranked in descending order as AM > E > Aq > HA (Figure 3A). A similar trend was noted for TFC: AM > Aq > E > HA (Figure 3B). The AM extract exhibited the highest TPC (213 ± 0.008 mg GAE/g DW) and TFC (138.1 ± 0.03 mg RE/g DW) of all extracts.

#### 3.1.2 Antioxidant potential

The result of the DPPH assay demonstrated the significant antioxidant potential of *P. glaucum* extracts. All extracts showed a significant dose-dependent inhibition of the free radicals, and maximum inhibition was observed by AM extract—60.7% at a concentration of 90 μg/mL (Figure 3C). The IC-_50_ of AM was recorded as 45.68 μg/mL—more statistically significant than other extracts (Figure 3D).

The result of reducing the power assay also demonstrated the significant antioxidant potential of *P. glaucum* extracts. AM extract showed the maximum absorbance, which correlates with the extract’s electron transfer ability, thus serving as a significant indicator of antioxidant potential (Figure 3E). All the extracts showed higher absorbance than the standard ascorbic acid. The EC-_50_ of AM extract was recorded as 38.50 μg/mL, while for ascorbic acid it was recorded as 45.60 μg/mL.

AM extract showed significant antioxidant potential in both assays (Supplementary Table S2).

#### 3.1.3 Anti-inflammatory potential

The albumin denaturation assay demonstrated the anti-inflammatory potential of *P. glaucum* extracts. Dose-dependent inhibition was observed for all extracts, with the AM extract showing the highest percentage inhibition, surpassing aspirin (Figure 3F). Similarly, IC-_50_ of AM extract showed more statistically significant results than aspirin: 34.09 μg/mL and 109.47 μg/mL, respectively (Figure 3G).

Proteinase inhibition assay also demonstrated the anti-inflammatory potential of *P. glaucum* extracts. Dose-dependent inhibition was observed for all extracts where AM extract exhibited higher inhibition than aspirin (Figure 3H). The IC-_50_ for the AM extract and aspirin were 51.36 μg/mL and 87.60 μg/mL, respectively, indicating the greater inhibitory potential of the AM extract than the positive control (Figure 3I).

Both assays indicated the AM extract as potentially anti-inflammatory Supplementary Table S2.

### 3.2 *In silico* evaluation of *Pennisetum glaucum* active compounds and their potential targets for treating rheumatoid arthritis

#### 3.2.1 *P. glaucum* active compound screening

Some 223 compounds were identified from *P. glaucum* GC-MS analysis. Active compounds were screened using various ADMET, drug-likeliness, and physiochemical parameters (Table 1). Based on these, 17 compounds were selected for further analysis**.**

**TABLE 1:** Parameters for the screening of active compounds.

Compound name	Code	PubChem CID	Lipinski’s rule of five	Physicochemical properties				Absorption			Toxicity				
				Molecular weight g/mol	HBA	HBD	TPSA Å^2^	Caco-2 permeability	Pgp-Substrate	HIA	HERG Blocker	H-HT	AMES	Acute toxicity	Carcinogenicity
o-Toluic acid, 2-butyl ester	C1	226914	Accepted	192.25	2	0	26.30	−4.335	No	Negative	Negative	Negative	Negative	Negative	Negative
Isobutyl methyl carbonate	C2	22556363	Accepted	132.16	3	0	35.53	−4.284	No	Negative	Negative	Negative	Negative	Negative	Negative
Methyl-3-(2,2-dichlorovinyl)-2,2-dimethyl-(1-cyclopropane)carboxylate,c andt	C3	94591	Accepted	223.10	2	0	26.30	−4.978	No	Negative	Negative	Negative	Negative	Negative	Negative
2-Methylamino-N-phenyl-acetamide	C4	541846	Accepted	164.20	2	2	41.13	−4.933	No	Negative	Negative	Negative	Negative	Negative	Negative
Benzenemethanol, 2-(2-aminopropoxy)-3-methyl-	C5	93285	Accepted	195.26	3	0	55.48	−4.914	No	Negative	Negative	Negative	Negative	Moderate	Negative
1-(5-Bicyclo [2.2.1]heptyl)ethylamine	C6	161455	Accepted	139.24	1	1	26.02	−4.811	No	Negative	Negative	Negative	Negative	Negative	Negative
Benzenesulfonamide, 2-nitro-N-phenyl-	C7	229600	Accepted	278.28	4	1	100.37	−4.644	No	Negative	Negative	Moderate	Negative	Negative	Moderate
3,4-Hexanediol, 2,5-dimethyl-	C8	552199	Accepted	146.23	2	2	40.46	−4.445	No	Negative	Negative	Negative	Negative	Negative	Negative
Pregnane-3,20-diamine, (3.beta.,5.alpha.,20S)-	C9	22213384	Accepted	318.54	2	2	52.04	−5.119	No	Negative	Moderate	Negative	Negative	Negative	Negative
Pentanoic acid, 2-hydroxy-4-methyl-, methyl ester	C10	62908	Accepted	146.18	3	1	46.53	−4.259	No	Negative	Negative	Negative	Negative	Negative	Negative
1-Propanamine, N1-methyl-2-methoxy	C11	541506	Accepted	103.16	2	1	21.26	−4.542	No	Negative	Negative	Negative	Negative	Negative	Negative
2-Piperidinone, 1-methyl-	C12	13603	Accepted	113.16	1	0	20.31	−4.456	No	Negative	Negative	Negative	Negative	Negative	Negative
dl-Alanine ethyl ester	C13	69236	Accepted	117.15	3	1	52.32	−4.88	No	Negative	Negative	Negative	Negative	Negative	Negative
sec-Butyl pentyl disulfide	C14	536233	Accepted	192.39	0	0	50.60	−4.252	No	Negative	Negative	Negative	Negative	Negative	Negative
5-Butyl-1,3-oxathiolan-2-one	C15	535042	Accepted	160.23	2	0	51.60	−4.475	No	Negative	Negative	Negative	Negative	Negative	Moderate
2,6-Lutidine 3,5-dichloro-4-dodecylthio-	C16	536613	Accepted	376.43	1	0	38.19	−4.744	No	Negative	Negative	Negative	Negative	Negative	Negative
Benzenethanamine, 2-fluoro-2′,4,5-trihydroxy-N-methyl-	C17	541604	Accepted	201.19	5	4	72.72	−5.031	No	Negative	Negative	Negative	Moderate	Negative	Negative

#### 3.2.2 Target prediction and network analysis

**FIGURE 4:**
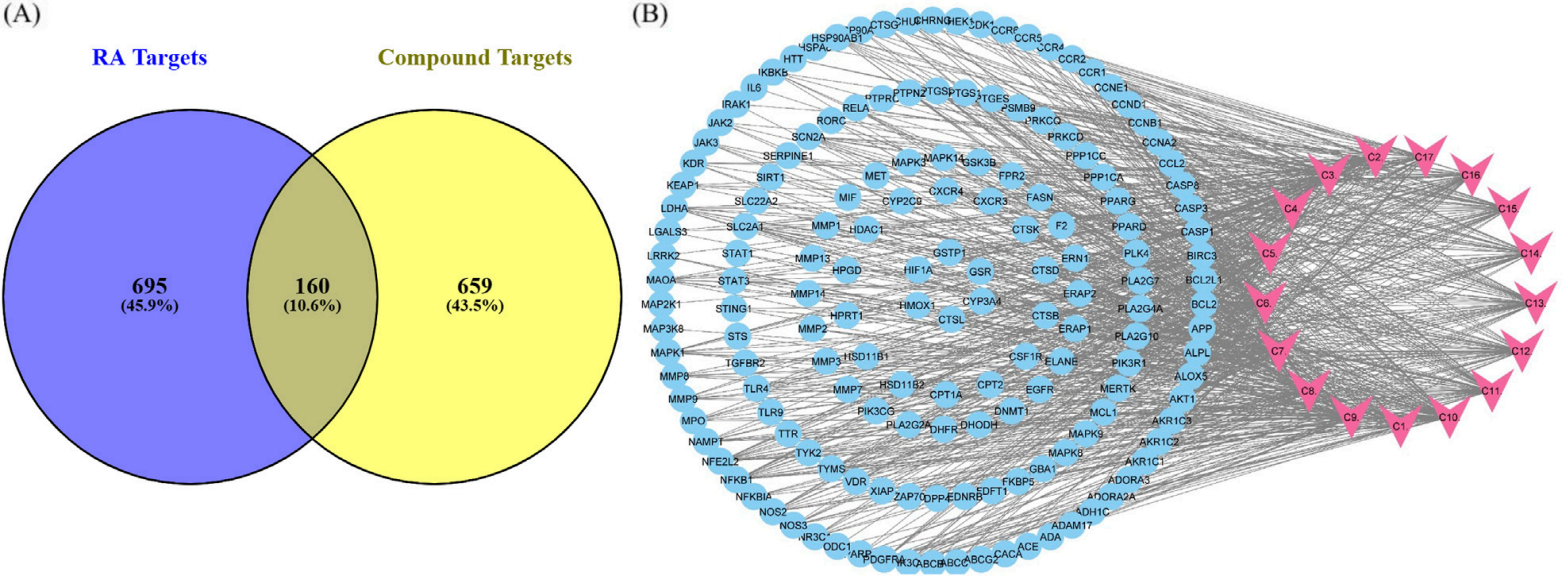
**(A)** Common targets between compounds and disease shown by a Venn diagram. **(B)** Compound–target pathway blue nodes represent the targets, while pink nodes represent the bioactive compound.

Potential targets of active compounds were identified, with 448 targets screened from the Swiss Target Prediction database and 594 from Super-PRED. A total of 819 targets was obtained after deduplication **(** Supplementary Figure S1A). CTD, GeneCard, OMIM, and the literature were employed to acquire 481, 412, 184, and 22 disease targets, respectively. A total of 855 disease targets were predicted after deduplication (Supplementary Figure S1B). Common targets of RA and compounds were predicted using a Venn diagram. A total of 160 anti-arthritic targets of *P. glaucum* were selected and considered as key therapeutic targets (Figure 4A).

Cytoscape was employed to construct a compound–target (C-T) network from 17 compounds and 160 common targets to comprehend the multi-target effect of *P. glaucum* in RA (Figure 4B). The C-T network had 177 nodes and 800 edges. The degree of 17 compounds was analyzed; the higher the degree, the higher the connectivity, where compound 9 (C9) had the highest degree of connectivity—60 followed by C7 (degree:58) and C10 (degree:57). Therefore, these compounds were considered key nodes, suggesting the multi-component therapeutic role of *P. glaucum* on RA. The degree of all the compounds, along with the MCC, MNC, betweenness, and closeness are displayed in Supplementary Table S3.

#### 3.2.3 Hub gene identification by the PPI network

**FIGURE 5:**
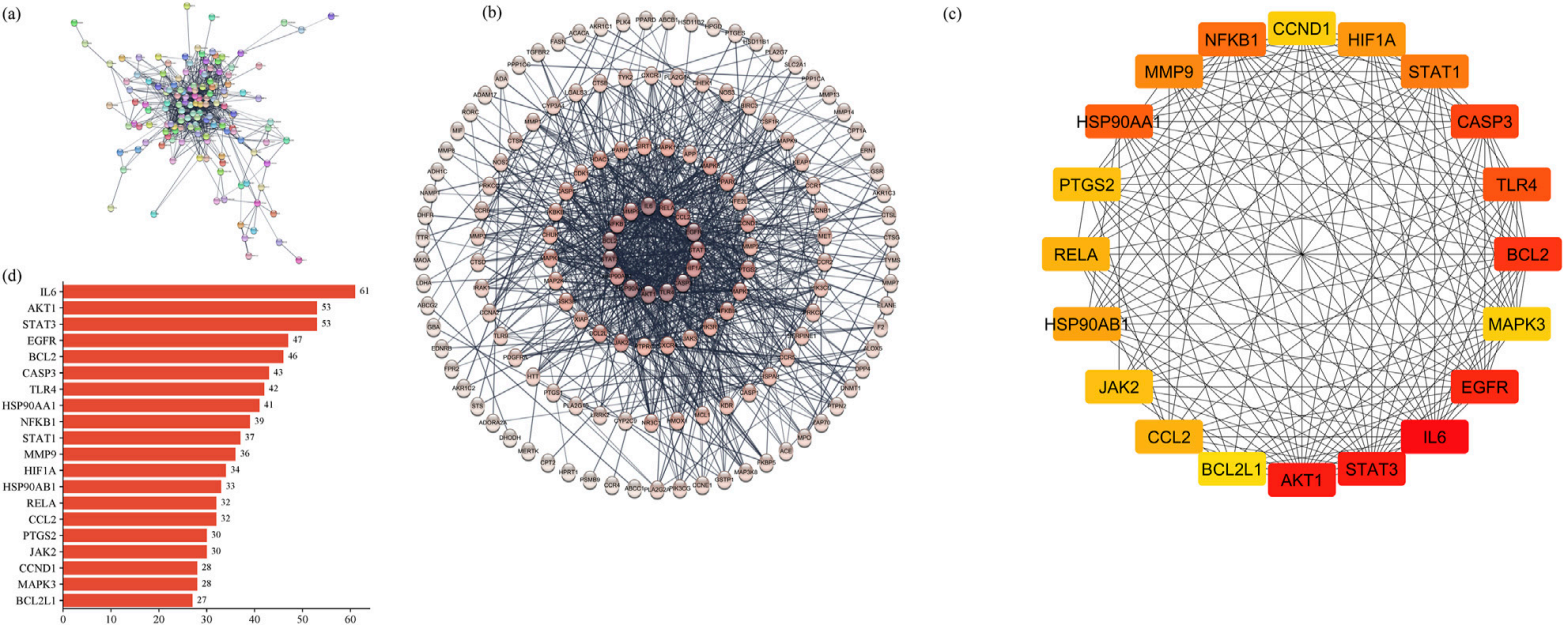
**(A)** PPI network of common 160 genes. **(B)** Degree sorted PPI network; darker color shows a higher degree. **(C)** Degree-based ranking of top 20 genes. **(D)** Bar plot of top 20 genes from the PPI network.

The STRING database was employed to construct the PPI network of 160 common targets. The PPI network had 949 edges (Figures 5A, B) and was exported to Cytoscape, where CytoHubba was employed to screen the top 20 hub genes based on degree connectivity (Figure 5C). The highest degree showed that the targets had stronger correlation among them and are the potential target. IL6, STAT3, AKT1, EGFR, BCL2, CASP3, TLR4, HSP90AA1, NFKB1, STAT1, MMP9, HIF1A, HSP90AB1, CCL2, RELA, JAK2, PTGS2, CCND1, MAPK3, and BCL2L1 showed the highest degree and were considered as the hub genes. The degree value of all these targets is shown in Figure 5D.

#### 3.2.4 Gene ontology and KEGG enrichment analysis

**FIGURE 6:**
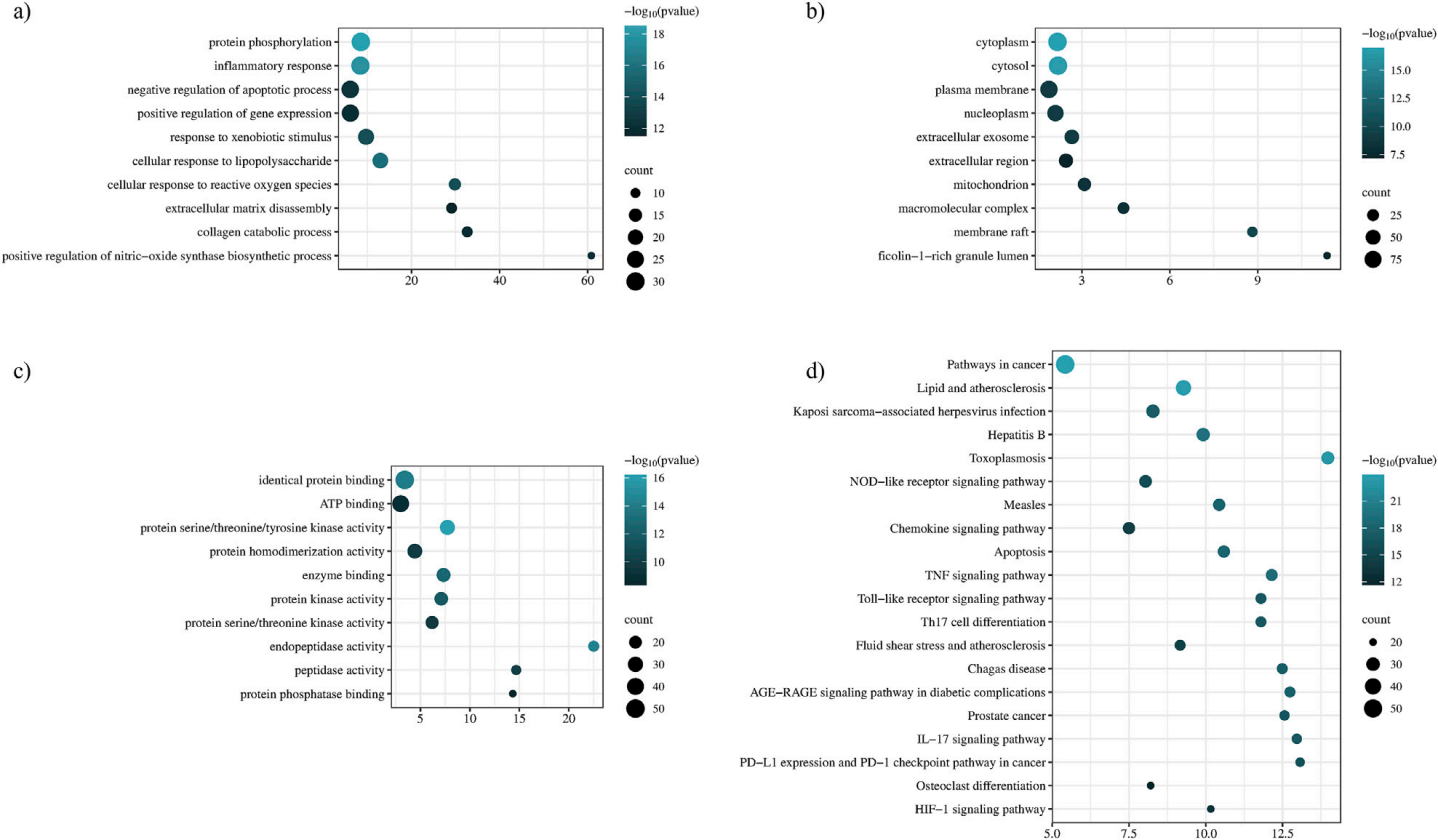
Functional annotation and enrichment analysis. **(A)** Gene ontology of BP, **(B)** gene ontology of CC, **(C)** gene ontology of MF, and **(D)** KEGG enrichment analysis.

Enrichment analysis and functional annotation revealed that *P. glaucum* targets were enriched in 518 BP, 74 CC, 123 MF, and 155 KEGG statistically significant pathways. The top ten enriched terms based on counts and *p*-value were visualized using a bubble plot (Figure 6). GO BP revealed that the targets of *P. glaucum* were enriched in protein phosphorylation, inflammatory response, the negative regulation of apoptotic process, extracellular matrix disassembly, collagen catabolism, and related processes (Figure 6A). GO CC analysis revealed that the targets were enriched in cytoplasm, cytosol, plasma membrane, nucleoplasm, and so forth (Figure 6B). GO MF analyses indicated that the targets were enriched in identical protein binding, ATP binding, protein serine/threonine/tyrosine kinases, protein kinase, protein serine/threonine kinases, and related activities (Figure 6C).

**FIGURE 7:**
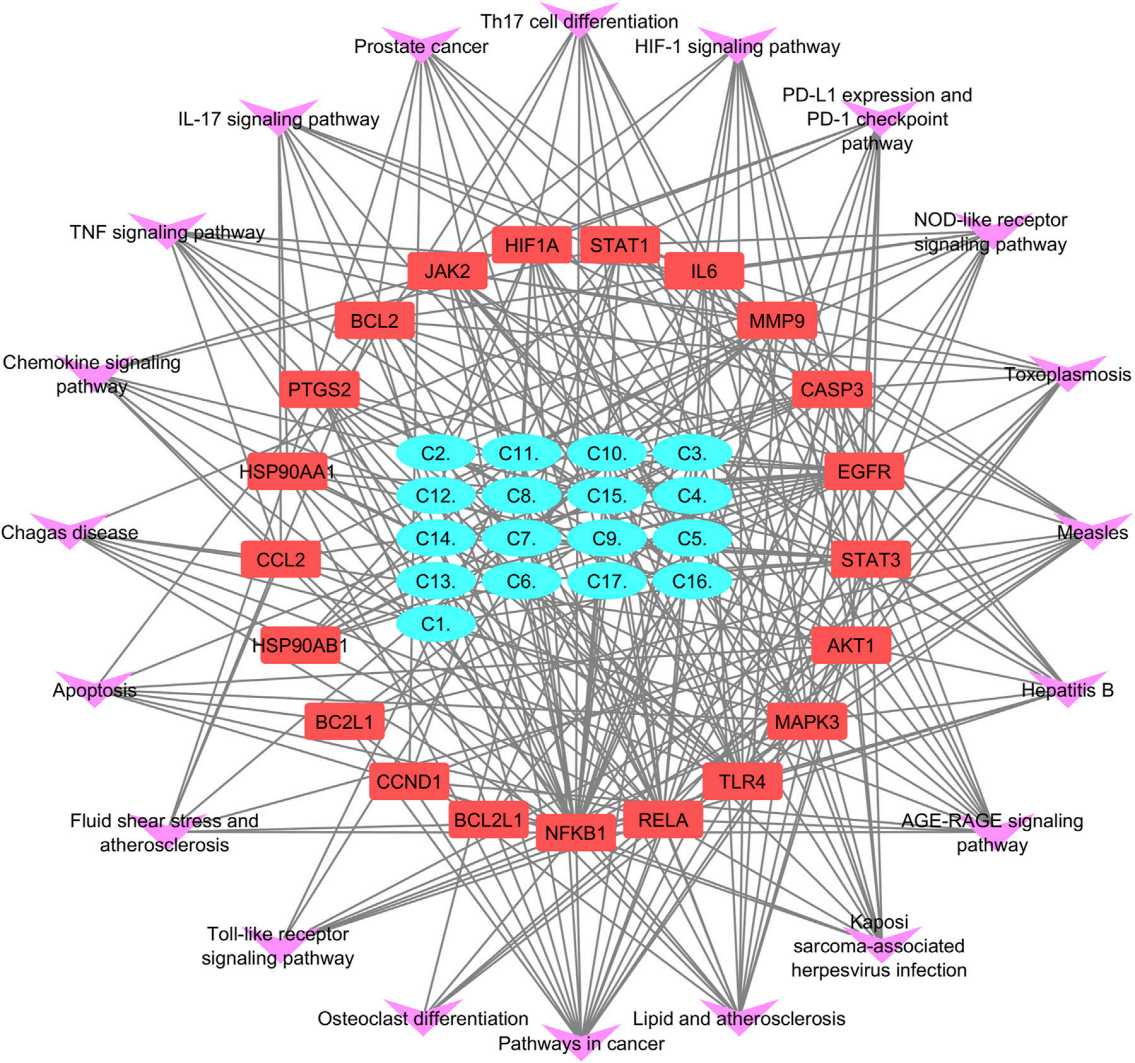
Compound–target-pathway network purple errors show pathways, red represents target gene, and cyan shows lead compounds of *Pennisetum glaucum*.

KEGG pathway exploration was conducted to investigate the most significant signaling pathway (Figure 6D). Notably, most of the genes were enriched in pathways in cancer, TNF-alpha signaling pathway, apoptosis, IL17 signaling pathway, Th17 cell differentiation, toll-like signaling pathway, HIF1 signaling pathway, and osteoclast differentiation (Table 2). The compound-target-pathway network in Figure 7 shows the enrichment of KEGG pathways and hub genes in association with the bioactive compounds.

**TABLE 2:** Top 20 hub gene-related compounds and targeted pathways.

Gene	Compound	Score	Pathways
IL6	C5	61	Pathways in cancer, TNF signaling, IL-17 signaling, toll-like receptor signaling, Th17 cell differentiation, NOD-like receptor signaling, and HIF-1 signaling pathway
STAT3	C1, C3, C6, C7, C9, C14, and C17	53	Pathways in cancer, Th17 cell differentiation, chemokine signaling, and HIF-1 signaling pathway
AKT1	C4	53	Pathways in cancer, TNF signaling, apoptosis, toll-like receptor signaling pathway, chemokine signaling, HIF-1 signaling pathway, and osteoclast differentiation
EGFR	C2, C3, C4, C5, C6, C7, C8, C9, C10, C12, C13, and C15	47	Pathways in cancer and HIF-1 signaling pathway
BCL2	C9 and C16	46	Pathways in cancer, apoptosis, NOD-like receptor signaling, and HIF-1 signaling pathway
CASP3	C7, C8, C10, C11, and C17	43	Pathways in cancer, TNF signaling pathway, apoptosis, and IL-17 signaling pathway
TLR4	C2,C3, C6, C9, C10, C12, C13, C14, C15, and C16	42	Toll-like receptor signaling pathway, NOD-like receptor signaling, and HIF-1 signaling pathway
HSP90AA1	C6, C9, C13, and C15	41	Pathways in cancer, IL-17 signaling pathway, Th17 cell differentiation, and NOD-like receptor signaling pathway
NFKB1	C1, C2, C3, C4, C5, C6, C8, C9, C10, C11, C12, C13, C14, C15, C16, and C17	39	Pathways in cancer, TNF signaling, apoptosis, IL-17 signaling, toll-like receptor signaling, Th17 cell differentiation, NOD-like receptor signaling, chemokine signaling, HIF-1 signaling pathway, and osteoclast differentiation
STAT1	C2, C7, and C10	37	Pathways in cancer, toll-like receptor signaling, Th17 cell differentiation, NOD-like receptor signaling, chemokine signaling pathway, and osteoclast differentiation
MMP9	C2, C4, C7, C10, C11, C12, and C14	36	Pathways in cancer, TNF signaling pathway, and IL-17 signaling pathway
HIF1A	C2, C3, C8, C10, C11, C12, C16, and C17	34	Pathways in cancer and Th17 cell differentiation
HSP90AB1	C3, C6, C8, C9, C11, C12, C13, and C15	33	Pathways in cancer, IL-17 signaling pathway, Th17 cell differentiation, and NOD-like receptor signaling pathway
CCL2	C4	32	TNF signaling pathway, IL-17 signaling pathway, NOD-like receptor signaling pathway, and chemokine signaling pathway
RELA	C3, C7, C8, C10, C13, and C15	32	Pathways in cancer, TNF signaling, apoptosis, IL-17 signaling, toll-like receptor signaling, Th17 cell differentiation, NOD-like receptor signaling, chemokine signaling, HIF-1 signaling, and osteoclast differentiation
JAK2	C3, C5, C8, and C10	30	Pathways in cancer, Th17 cell differentiation, and chemokine signaling pathway
PTGS2	C1, C7, C8, C10, C13, C14, C15, and C16	30	Pathways in cancer, TNF signaling pathway, and IL-17 signaling pathway
CCND1	C13	28	Pathways in cancer
MAPK3	C10	28	Pathways in cancer, TNF signaling pathway, apoptosis, IL-17 signaling pathway, toll-like receptor signaling pathway, Th17 cell differentiation, NOD-like receptor signaling pathway, chemokine signaling pathway, HIF-1 signaling, and osteoclast differentiation
BCL2L1	C16	27	Pathways in cancer, apoptosis, and NOD-like receptor signaling pathway

#### 3.2.5 Microarray data analysis

**FIGURE 8:**
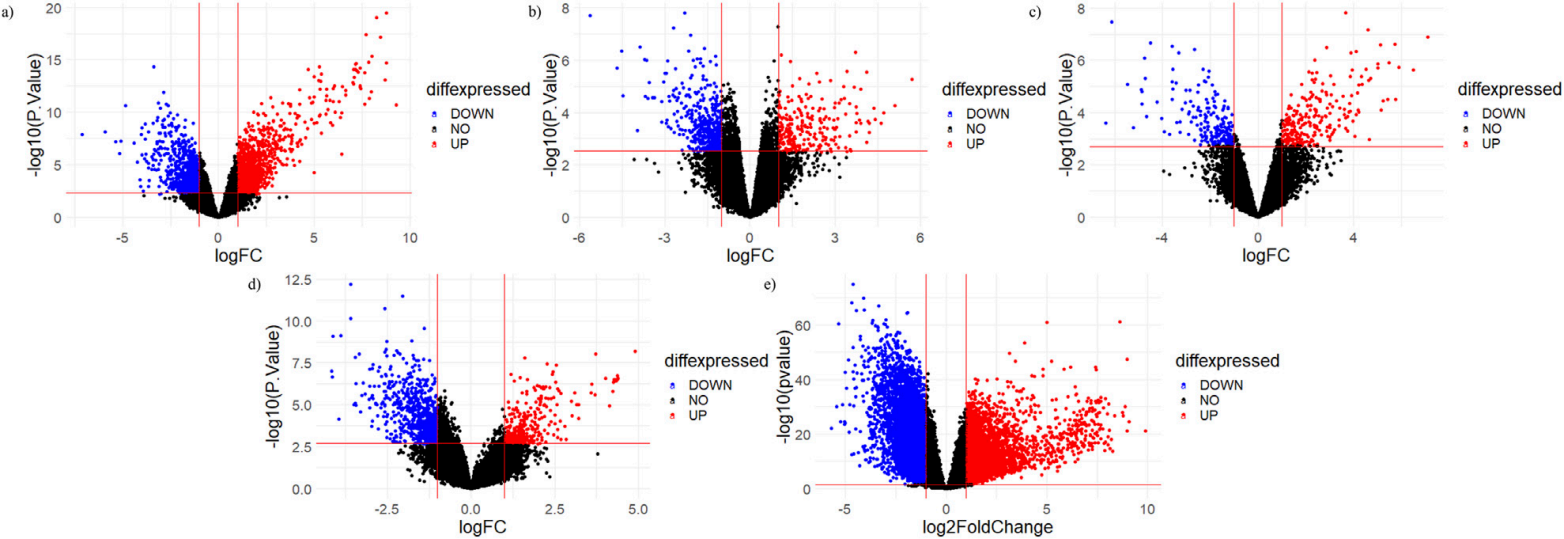
Volcano plots of micro array datasets. Red dots show upregulated genes, while blue dots show downregulated genes: **(A)** GSE55235, **(B)** GSE12021, **(C)** GSE 1919, **(D)** GSE21959, and **(E)** GSE89408 datasets.

The top 20 shortlisted hub genes were further validated by microarray data analysis (Figure 8). Five microarray datasets of RA and healthy control were selected to identify whether the selected hub genes were upregulated in RA. By concentrating on upregulated genes, we ensured a targeted and reliable strategy to identify the potential targets. Of the 20 hub genes, only nine were upregulated in these datasets: JAK2, STAT1, PTGS2, IL-6, MMP9, TLR4, CASP3, HIF1A, and CCL2 (Table 3). STAT1 was upregulated in all five datasets. MMP9 and JAK2 were upregulated in two datasets: GSE55235 and GSE89408. IL-6 was also upregulated in two datasets: GSE21959 and GSE89408. PTGS2, HIF1A, CCL2, CASP3, and TLR4 were upregulated in GSE89408. The nine upregulated genes were then selected for molecular docking.

**TABLE 3:** Detail of microarray datasets.

Dataset	Platform	Total	Control	Affected	Source	Upregulated	Downregulated
GSE55235	GLP96	30	10	10	Synovial	1058	781
GSE12021	GPL96	31	9	12	Synovial	254	457
GSE1919	GLP91	15	5	5	Synovial	245	191
GSE21959	GL04133	36	18	18	Synovial	338	741
GSE89408	GPL1115	218	23	93 (established)	Synovial	4,167	5,008

#### 3.2.6 Molecular docking

**FIGURE 9:**
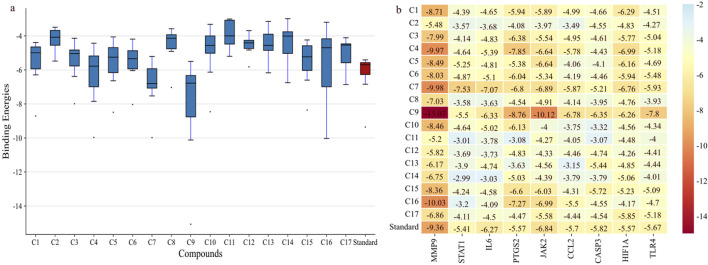
Binding affinities of shortlisted proteins with 17 compounds. **(A)** Boxplot showing that C9 and C7 have the best binding affinity among all the lead compounds; the red bar shows the binding affinity of different standards against each target. **(B)** Heatmap showing the binding affinities of proteins with the lead compounds.

Molecular docking was performed for nine hub genes with 17 active compounds. Docking analysis predicted strong interactions between the binding pocket of proteins and active compounds, as shown in the heatmap in Figure 9B. The findings were compared with the FDA approved standard drugs: fludarabine phosphate (for STAT1), fostamatinib (for JAK2), minocycline (for MMP9), nifulmic acid (for PTGS2), bazedoxifene (for IL-6) chondroitin 4-sulfate (for CCL2), aspirin (for CASP3), vadadustat (for HIF1A), and cyclobenzaprine (for TLR4). C7 and C9 exhibited significantly higher binding affinities for target genes, as illustrated by box plot (Figure 9A).

**FIGURE 10:**
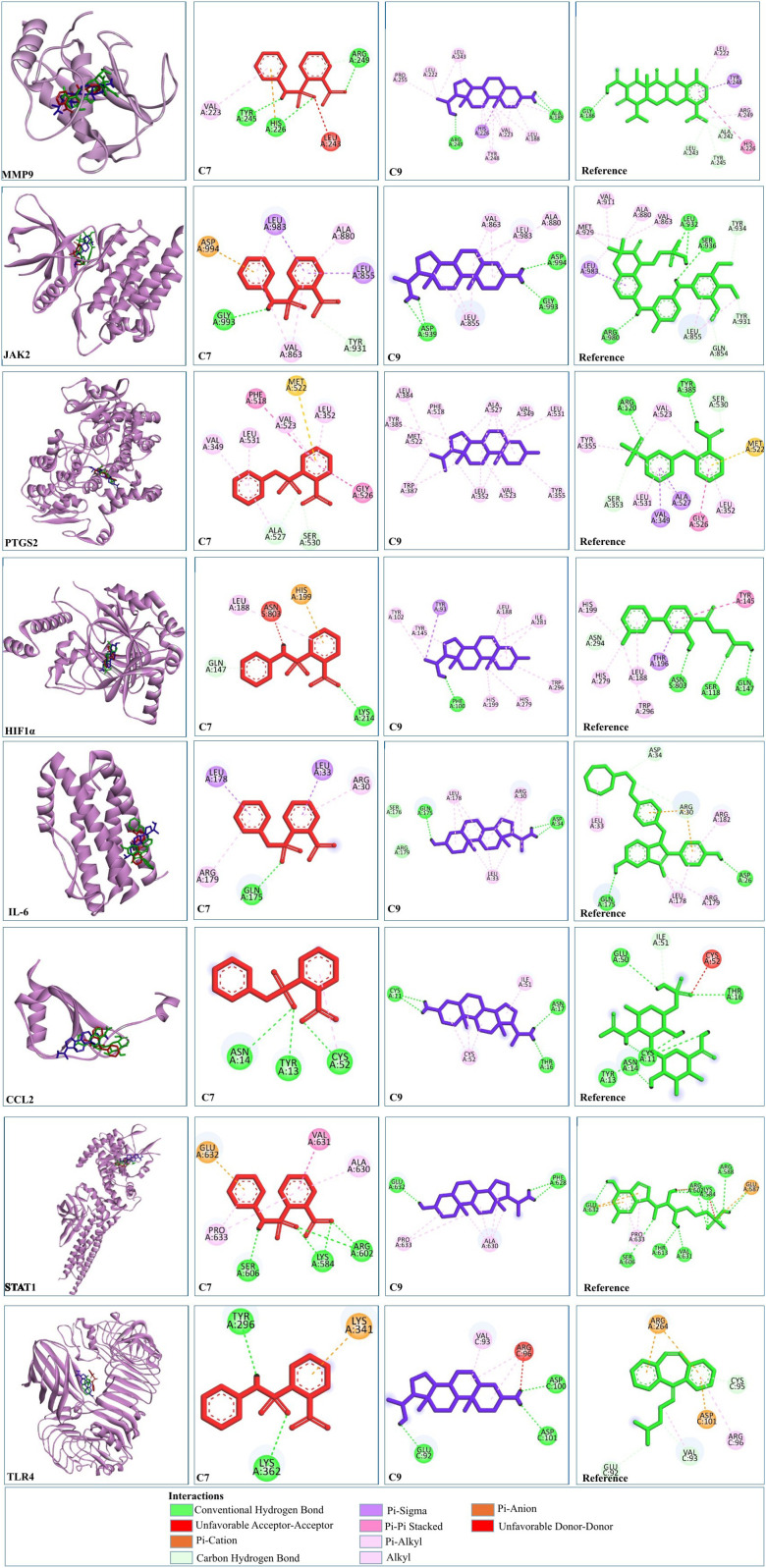
2D and 3D visualization of docked complexes of C7, C9, and reference with the shortlisted targets—MMP9, JAK2, PTGS2, HIF1A, IL-6, CCL2, STAT1, and TLR4. Red depicts C7, blue represents C9, and green represents the reference structure. Key indicates the types of interaction between compounds and targets.

C9 showed much stronger binding affinities than the reference drugs, with values of −15.07, −5.5, −6.33, −8.76, −10.12, −6.78, −6.56, −6.26, and −7.8 kcal/mol for MMP9, STAT1, IL-6, PTGS2, JAK2, CCL2, CASP3, HIF1α, and TLR4, respectively. The reference drugs (minocycline, fluradibine phosphate, bazedoxifene, niflumic acid, fostamatinib, chondroitin 4-sulfate, aspirin, vadadustat, and cyclobenzaprine) showed lower affinities, with values of −9.36, −5.41, −6.27, −5.57, −6.84, −5.7, −5.82, −5.57, and −5.67 kcal/mol for the same targets. Similarly, C7 demonstrated higher binding affinities for eight targets, with values of −9.98, −7.53, −7.07, −6.8, −6.89, −5.87, −6.76, and −5.93 kcal/mol for MMP9, STAT1, IL-6, PTGS2, JAK2, CCL2, HIF1A, and TLR4, respectively. These findings confirm the strong binding capabilities of C7 and C9 to target proteins, outperforming the references. Each drug candidate demonstrated significant interactions with receptor proteins (Supplementary Figures S2–10). C7 bound stably with the MMP9 binding pocket and formed H-bonds with Tyr245, His226, and Arg249 and hydrophobic interaction with Val223; the same interaction pattern was observed with the reference drug. However, C9 formed H-bonds with Arg249 and Ala189 and hydrophobic interactions with His226, Val223, Leu1s88, Tyr248, Pro255, Leu222, and Leu243. The common interacting residues between the standard and C9 were Tyr245, His226, Arg249 and Arg249, His226, Leu243, Leu222, and Tyr248. Figure 10 shows the binding interaction of the top eight targets with C7, C9, and the references. Supplementary Table S4 displays the detailed interaction of all the ligands and proteins in comparison with the reference drugs along with the binging affinities and inhibition constant (Ki).

#### 3.2.7 Molecular dynamic simulations

Molecular dynamic (MD) simulation was conducted to elucidate the stability of protein–ligand interactions and assess protein structural flexibility within the docked complexes. Complexes involving MMP9, JAK2, PTGS2, and HIF1α demonstrated consistent stability during the initial 30 ns period, thus warranting extension to 100 ns.

**FIGURE 11:**
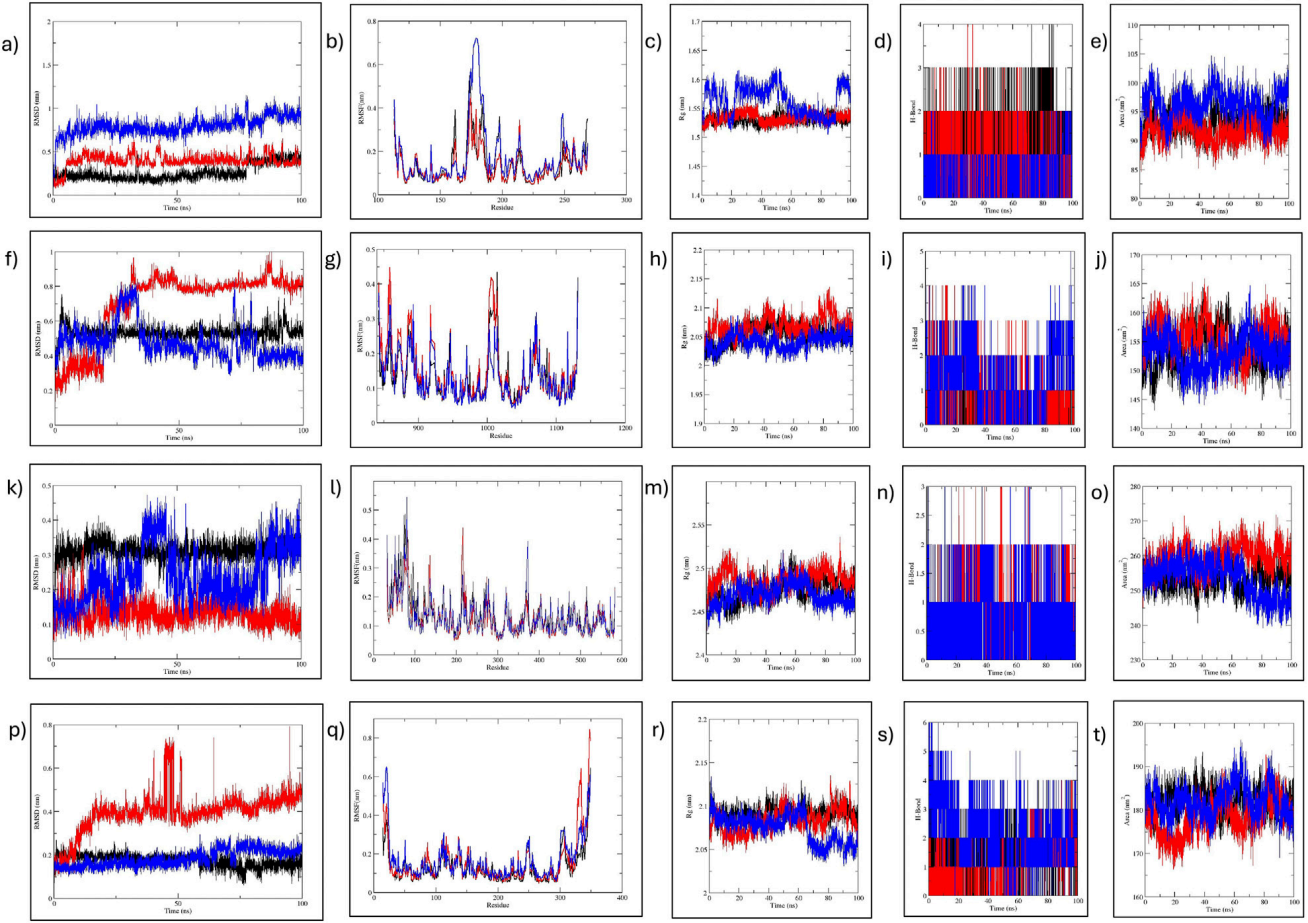
MD simulation analysis of MMP9, JAK2, PTGS2, and HIF1α. **(A–E)** MMP9-C7, C9, and reference analysis: **(A)** RMSD, **(B)** RMSF, **(C)** RoG, **(D)** H-bond, and **(E)** SASA analysis. **(F–J)** JAK2-C7, C9, and reference analysis: **(F)** RMSD, **(G)** RMSF, **(H)** RoG, **(I)** H-bond, and **(J)** SASA analysis. **(K–O)** PTGS2-C7, C9, and reference analysis: **(K)** RMSD, **(L)** RMSF, **(M)** RoG, **(N)** H-bond, and **(O)** SASA analysis. **(P–T)** HIF1a-C7, C9, and reference analysis: **(P)** RMSD, **(Q)** RMSF, **(R)** RoG, **(S)** H-bond, and **(T)** SASA analysis. Black represents C7; red, C9; and blue the reference.

First, RMSD values were calculated to estimate the structural stability of docked complexes. Throughout the 100 ns MD simulation, RMSD values for all complexes remained stable (Figure 11). For MMP9 protein, C7 complex exhibited the most stable RMSD, with an average value of 0.26 nm and experiencing minor fluctuations after 75 ns. This demonstrated greater stability than the C9 complex (0.396 nm). Both compounds showed better RMSD values than the reference (0.792 nm). For JAK2 and HIF1A, C7 displayed a very stable RMSD without significant fluctuations, outperforming both C9 and reference. The JAK2-C9 complex showed an increased trend after 25 ns. The JAK2–Reference complex showed a fluctuation at 25 ns and then stabilized with minor fluctuations throughout the 100 ns. HIF1A-C9 showed an increasing trend with slight fluctuations before 50 ns, then stabilized with minimal fluctuations until 100 ns. The HIF1A–Reference showed a comparable RMSD to C7. For PTGS2, both the C9 and C7 complexes exhibited a very stable RMSD. PTGS2–Reference showed slight fluctuations at 38 ns and 83 ns, but its average RMSD remained within the acceptable range at 0.239 nm (Table 4).

**TABLE 4:** Average values of RMSD, SASA, RoG, and H-Bonds formed during MD simulation of MMP9, JAK2, and PTGS2 with C7, C9, and R.

MD analysis	MMP9-C7	MMP9-C9	MMP9-R	JAK2-C7	JAK2-C9	JAK2-R	PTGS2-C7	PTGS2-C9	PTGS2-R	HIF1α-C7	HIF1α-C9	HIF1α-R
RMSD	0.260	0.396	0.792	0.532	0.703	0.478	0.313	0.134	0.239	0.175	0.395	0.184
RMSF	0.138	0.130	0.174	0.131	0.146	0.126	0.139	0.128	0.131	0.127	0.154	0.161
RoG	1.531	1.534	1.564	2.054	2.068	2.041	2.474	2.486	2.469	2.091	2.083	2.074
SASA	93.33	91.69	96.45	153.8	155.5	153.1	253.3	259.4	253.4	182.1	178.0	182.0
H-Bond	4	4	2	3	4	5	3	3	3	4	4	6

RMSF for the complexes were analyzed to assess flexibility at the residue level (Figure 11). For MMP9, minimal fluctuations were observed for both C7 and C9, while MMP9–Reference fluctuated slightly residues 173–184 at the C-terminus. The flexibility in all MMP9 complexes was noted in β-strands, indicating the dynamic nature of the binding pocket as it adapts to the bound ligand. In the case of JAK2, C7 and reference had comparable fluctuations while C9 had higher fluctuation at the same residues: 1006-1015 located in the protein kinase domain. Nonetheless, JAK2 complexes exhibited minimal fluctuation. For PTGS2, reference showed more fluctuation than the compounds, particularly at residues 81 and 373. PTGS2–C7 and –C9 showed stable RMSF, with C9 fluctuating at 83 residues in the helical region. For HIF1A, C7 and reference showed minimal fluctuation, whereas C9 fluctuated at the C-terminal residues 347 and 349. Overall, all complexes exhibited acceptable residue fluctuations, demonstrating high structural stability and rigidity (Table 4).

The compactness of the protein structure was analyzed by RoG, revealing that all complexes were stable (Figure 11). For MMP9, the RoG of the reference complex fluctuated between 1.5 and 1.6 nm, while the RoGs for MMP9–C7 and–C9 were nearly identical, indicating that both compounds preserved the MMP9 conformation much more stably than the reference. For JAK2, C9 and C7 RoGs were comparable to that of reference, showing that both compounds stabilized the protein effectively. Similarly, PTGS2–C9 and –C7 exhibited better RoG values (2.474 and 2.486 nm, respectively) than the reference (2.469 nm). The RoG of the HIF1α reference complex fluctuated 2.05–2.1 nm, while C7 and C9 had comparable RoG values, indicating that both compounds maintained the protein conformation better than the reference. Overall, the RoG data indicated that all complexes of C7 and C9 were structurally compact, with improved folding behavior compared to the reference complexes.

The H-bond analysis determined the stability of complex interactions. For MMP9, both C9 and C7 formed four H-bonds, while the reference formed two H-bonds. For JAK2, the reference formed five H-bonds while C7 and C9 formed three and four H-bonds, respectively. For PTGS2, all complexes formed three H-bonds. For HIF1α, both C7 and C9 formed four H-bonds, while reference formed six H-bonds (Figure 11).

Estimating SASA values is beneficial for predicting the protein-interactable surface with the solvent (Figure 11). For MMP9, the reference SASA ranged 90–100 nm^2^, while for C7 and C9 it ranged 90–95 nm^2^. For JAK2, C7 ranged 150–155 nm^2^, while C9 and reference showed fluctuation at approximately 155 nm^2^, suggesting similar surface accessibility for both. For PTGS2, all complexes displayed similar surface accessibility until 60 ns. Thereafter, reference and C7 showed a decrease, while C9 remained stable with minor fluctuations. In the case of HIF1α, C7 remained stable at 180–185 nm^2^, and C9 fluctuated 170–185 nm^2^. The reference fluctuated 185–190 nm^2^.

#### 3.2.8 PASS prediction of biological activity

The identification of molecular interactions between compounds and their targets is important for *in silico* and toxicity analysis. The PASS tool uses structure–activity relationship analysis (SAR) to predict various biological targets, hence reducing the possibility of failure while performing *in vitro* and wet-lab testing. The PASS tool showed 1130 and 482 biological activities of C7 and C9, respectively, primarily focusing on anti-inflammatory signaling pathways and potential targets for RA. The tool successfully identified C7 and C9 as potential JAK2 (Pa = 0.267 for C7 and Pa = 0.322 for C9) and prostaglandin (Pa = 0.279 for C7 and Pa = 0.226 for C9) inhibitors. C7 was reported as a non-steroid anti-inflammatory agent (Pa = 0.188) with an anti-inflammatory Pa of 0.206 and was also reported to inhibit IL6 (Pa = 0.155), CC chemokine 9 and 10 receptor (Pa = 0.119), CXC chemokine 2 and 1 receptor (Pa = 0.051), and transcription factor NFKβ (Pa = 0.282). C9 was reported as steroidal anti-inflammatory (Pa = 0.308) and an antagonist of IL-2 and 10 (Pa = 0.448), interferons (Pa = 0.159)—particularly interferon gamma (Pa = 0.153)—and transcription factor NFKα (Pa = 0.229). C7 was also reported to inhibit MMPs (Pa = 0.053), particularly MMP1, 2, and 9 (Pa = 0.030, 0.054 and 0.032 respectively). C7 was also recognized as a collagenase inhibitor (Pa = 0.138). All biological activities of C7 and C9 predicted by the PASS are detailed in Supplementary File S2. These diverse arrays of biological activities suggest that C7 and C9 hold promise as multifaceted treatment candidates for RA.

## 4 Discussion

RA is a complex autoimmune condition characterized by hyperinflammatory and abnormal proteolytic activity. The limitations of current therapeutic options have demanded a shift toward medical plants, leading to a move from traditional methods to computer-based assessments with the aim of improving drug discovery, such as network pharmacology. These can offer insights into compound interactions and address concerns about research costs and drugs that lack ADME qualities (Cai et al., 2020).

Research on natural-compounds-based therapeutics has increased due to their safer profile in the efficient management of the various pathologies (Jha et al., 2023). *Pennisetum glaucum* has been reported to play a significant role in bone metabolism and osteoimmunology (Nani et al., 2020). This highly nutritious cereal crop has garnered significant global attention, leading the UN Food and Agriculture Organization to recognize 2023 as the “International Millet Year” (Harish et al., 2024). Acknowledged as a “nutri-cereal” by the Government of India, pearl millet stands out for its elevated nutrient content, particularly in unsaturated and omega-3 fatty acids (Deka et al., 2024), and it has been reported that these fatty acids modulate the pathogenesis of arthritis (Kostoglou-Athanassiou et al., 2020). The phytochemical diversity of pearl millet, including polyphenols, flavonoids, alkaloids, saponins, tannins, and anthraquinones, underpins its various pharmacological properties, such as antioxidative, antimicrobial, antidiabetic, antihypertensive, anti-inflammatory, anticholesterolemic, and bone protecting effects, offering protection against numerous disorders (Ahmed et al., 2023). The aim of this study is to identify the potential bioactive compounds of *P. glaucum* to alleviate RA.

This study accessed the phenolic, flavonoid, antioxidant, and anti-inflammatory properties of *P. glaucum* extracts. The AM extract showed the highest levels of phenols and flavonoids, likely due to the effectiveness of the acidified solvent in extracting maximum polyphenols that resulted in stronger antioxidant and anti-inflammatory potential. Our results are more or less similar to previous studies (Siroha et al., 2016). Chethan and Malleshi (2007) reported that millet polyphenols are more stable in acidic conditions than alkaline. Consequently, the acidified extract underwent GCMS analysis to generate a compound library. Compounds that met all ADMET criteria to ensure that they were non-toxic, non-mutagenic, and orally effective were selected for further investigation, adopting an approach similar to Maradesha et al. (2023). The study utilized a network pharmacology approach to identify 160 anti-RA targets of *P. glaucum*. These were subjected to PPI and KEGG enrichment analysis where pathways such as TNF, IL-17, TH-17, toll-like receptor, NOD-like receptor, chemokine signaling, apoptosis, and osteoclast differentiation exhibited significant enrichment; dysregulation of these signaling pathways plays a pivotal role in RA (Baier et al., 2003). Compound–target network analysis revealed that all compounds exhibited strong interactions, especially C7 and C9, indicating potential anti-RA properties and validating our hypothesis that *P. glaucum* can effectively alleviate RA. The top 20 hub genes were selected from a PPI network by employing the degree algorithm of cytohubba. All 20 genes play a crucial role in RA pathogenesis, but to ensure their clinical relevance, they were assessed across five microarray datasets. Only nine genes were upregulated, revealing their significant role in disease progression and severity; a similar approach was utilized by Sadaqat et al. (2023). This finding was corroborated by docking nine genes with 17 compounds, which indicated significant docking energies (Figure 6). Importantly, C7, C9, and C4 emerged as particularly promising candidates in contrast to the reference drugs—minocycline was reported to cause hyperpigmentation (Fay et al., 2008) and other severe side effects in RA (Suresh et al., 2004), niflumic acid has reported nephrotoxic effect (Aronson JKBT-MSE of D and 16th, 2016), fostamatinib is associated with cardiovascular risk (Chen Y. et al., 2021), and vadadustat has reported to increase thrombosis events (Chen H. et al., 2021). Furthermore, ADMET profiles of the reference drugs revealed that they all exhibited some form of toxicity, including hepatotoxicity, acute toxicity, and carcinogenicity, except vadadustat (**S2**).

C7 and C9 complexes, which showed better binding energy than the reference drugs, were subjected to MD simulation. Initially, all complexes underwent a 30 ns MD simulation, during which complexes of STAT1 and TLR4 were unstable. In the case of IL-6, C7 initially deviated from the binding pocket but returned by the end of the 30 ns MD simulation, while both C9 and the reference left the binding pocket. Similar observations were made for CCL2 and thus were not extended further (Supplementary Figure S11). However, MMP9, JAK2, PTGS2, and HIF1A complexes were extended to 100 ns, demonstrating stability as strong inhibitors within the binding pocket of proteins. C7, an aromatic sulfur-containing compound, has also been reported in the GCMS analysis of soya bean extract (Chukeatirote et al., 2017), while its derivatives have been reported in the extracts of various medicinal plants (Ejiofor et al., 2022; Al-Rajhi et al., 2022). C9, an alkaloid steroid compound, is a pregnane derivative and has also been reported in many medical plants (Kadam and Gaykar, 2018; Nadar, 2015) and as exhibiting potential biological activities (Si et al., 2022). Finally, the PASS analysis confirmed the biological potential of C7 and C9 as promising targets for RA treatment, showing higher Pa values for inhibiting cyclooxygenase (responsible for pain and inflammation), MMPs (involved in cartilage destruction and bone erosion), JAK/STAT (crucial for aberrant signaling pathways), cytokines and chemokines (pivotal in RA pathogenesis), and specific inhibitors of the TCA cycle (facilitating the conversion of lactate in hypoxic environments, leading to increase inflammation) (Cai et al., 2020). The collective results of MD simulation and PASS analyses highlight C7 and C9 as potent inhibitors for MMP9, JAK2, PTGS2, and HIF1α proteins, warranting further exploration in therapeutic development.

These four genes play an intricate role in RA pathogenesis and collectively orchestrate a complex network of interactions. MMP9 secreted by FLS and neutrophiles lead to the degradation of cartilage/bone collagen and ECM membrane, facilitating the stimulation of angiogenic factors and cytokines in RA (Bian et al., 2023). JAK2, stimulated by various cytokines, stimulates angiogenesis (Cheng et al., 2020), hinders fibroblast apoptosis (Krause et al., 2002), induces MMP expression, and stimulates osteoblastic activity in RA. PTGS2 stimulated the synthesis of PEG2, which modulates bone resorption, pain, and inflammation and is associated with MMP secretion in RA (Ozen et al., 2019). HIF1a induced hypoxic environment and increased glycolysis which stimulates cytokines, VEGF, and rapid proliferation of RA-FLS (Ahmed et al., 2023). Therefore, the ability of C7 and C9 to interact with HIF1α, JAK2, PTGS2, and MMP9 suggests a multi-target approach that could effectively regulate inflammation, RA-FLS, immune response, and tissue degradation associated with RA.

This multi-faceted strategy holds great promise for managing RA by addressing various aspects of the disease’s complex pathophysiology. In previous studies on protein–ligand systems, Alsaif et al. (2024) and Yu et al. (2024) utilized molecular docking analysis, identifying significant binding affinities between target proteins and their respective compounds. In contrast, our research employed a more comprehensive set of techniques, incorporating molecular docking, MD simulation, and PASS analysis. This multifaceted approach allowed a thorough understanding of protein–ligand interactions and provided greater confidence in the results. Our study lays the groundwork for considering *P. glaucum* compounds as a potential treatment for RA.

## 5 Conclusion

This study identified the plant-based bioactive compounds of *Pennisetum glaucum* against RA by *in vitro* and *in silico* approaches. It concluded that *P. glaucum* and its bioactive compounds hold significant anti-rheumatic potential. *In vitro* analysis revealed a significant antioxidant and anti-inflammatory potential of *P. glaucum* extracts, which are crucial for anti-rheumatic therapies. *In silico* analysis, utilizing CADD techniques such as network pharmacology, microarray validation, molecular docking, and MD simulations, identified the potential bioactive compound and targets that influence biological processes such as inflammation, apoptosis, and collagen degradation, as well as pathways like TNF signaling, HIF1 signaling, and osteoclast differentiation, which are crucial in managing RA. Two bioactive compounds—C7 and C9—were identified with therapeutic potential against the key RA targets MMP9, JAK2, PTGS2, and HIF1A. The insights gained from this study could guide the future application and development of *P. glaucum* as a treatment for RA.

## Data Availability

The datasets presented in this study can be found in online repositories. The names of the repository/repositories and accession number(s) can be found in the article/Supplementary Material.
