# Supplementary material for: Evaluation of the inhibitory mechanism of Pennisetum glaucum (pearl millet) bioactive compounds for rheumatoid arthritis: an in vitro and computational approach
Source: Front Pharmacol. 2024 Nov 21;15:1488790. doi: 10.3389/fphar.2024.1488790 (PMC11617165; doi:10.3389/fphar.2024.1488790)
Supplement: Supplementary file 2 [file DataSheet1.docx]

**Evaluation of Inhibitory mechanism of *Pennisetum glaucum* (Pearl Millet) Bioactive Compounds for Rheumatoid Arthritis: An In Vitro and Computational Approach**

**Maria Sharif ^1^, Peter John ^1*^ , Attya Bhatti ^1^, Rehan Zafar Paracha^2^, Abid Majeed^3^**

^1^ Department of Biomedicine, Atta-ur-Rahman School of Applied Biosciences (ASAB), National University of Sciences & Technology (NUST), Islamabad, Pakistan.

^2^  School of Interdisciplinary Engineering & Sciences (SINES), National University of Sciences & Technology (NUST), Islamabad, Pakistan.

^3^ Crop Sciences Institute, National Agriculture Research Center (NARC), Park Road, Islamabad, Pakistan.

*** Correspondence:** pjohn@asab.nust.edu.pk ; Tel.: +92-051-90856151.


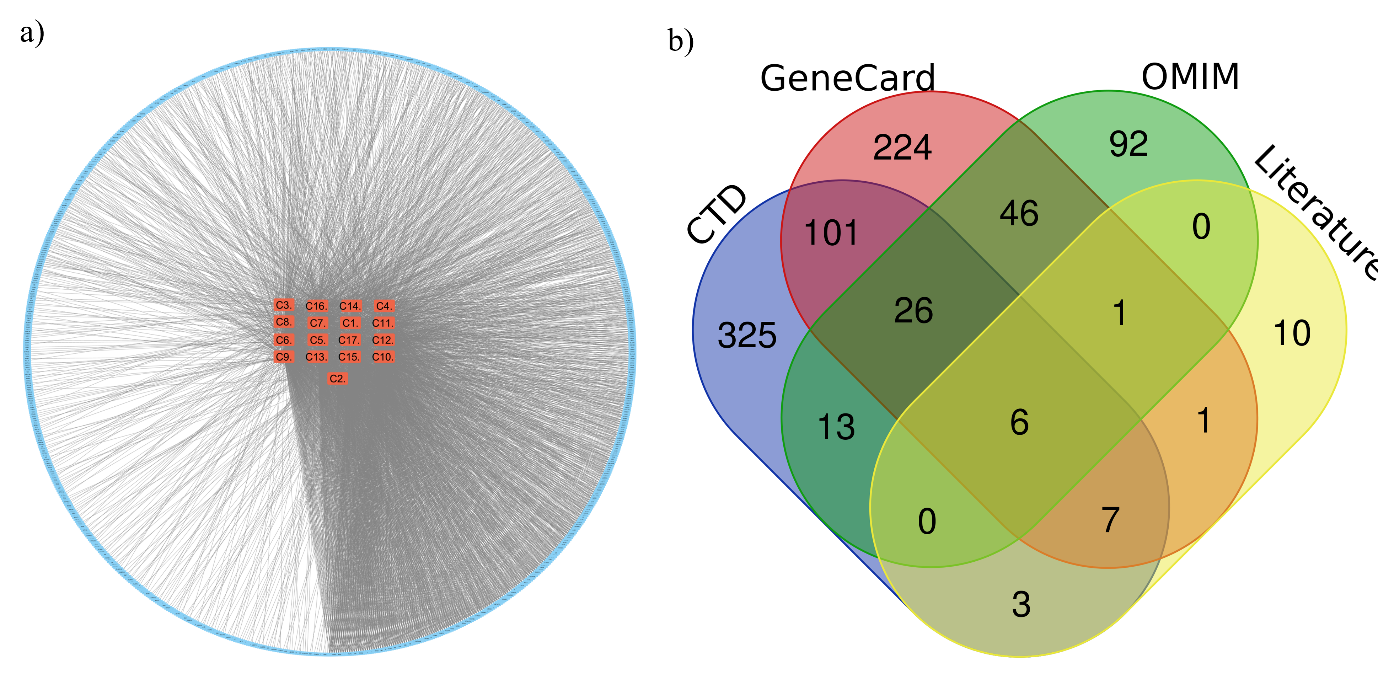
Figure 1

Figure 1 Target prediction A) Targets of P. glaucum active compounds from Swiss Target Prediction and Super-PRED, Blue color represents the targets while red color represents the compounds, B) RA targets retrieved from different sources.


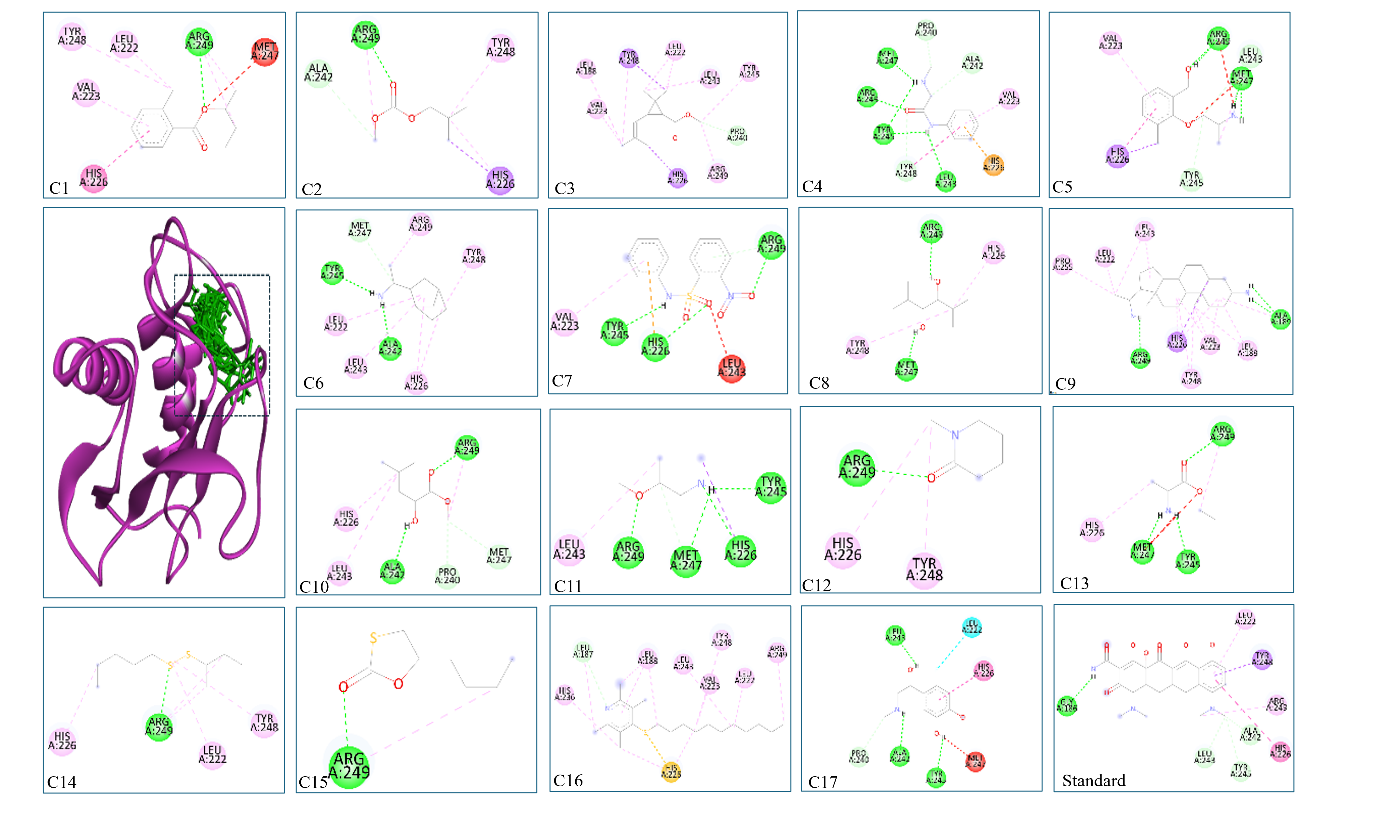
Figure 2 MMP9

Figure 2 2D visualization of MMP9protein with lead compounds and reference.


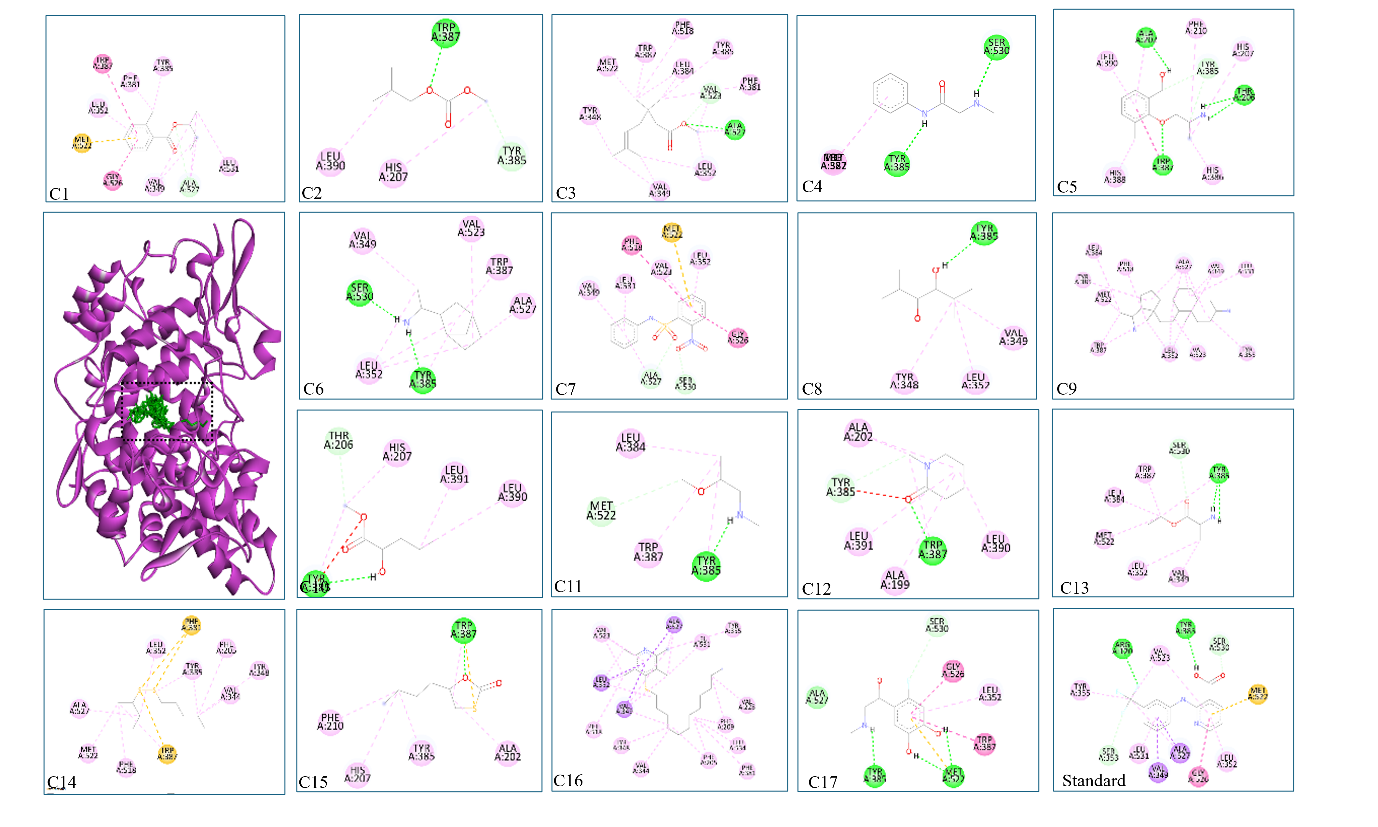
Figure 3 PTGS2

Figure 3 2D visualization of PTGS2 protein with lead compounds and reference.

Figure
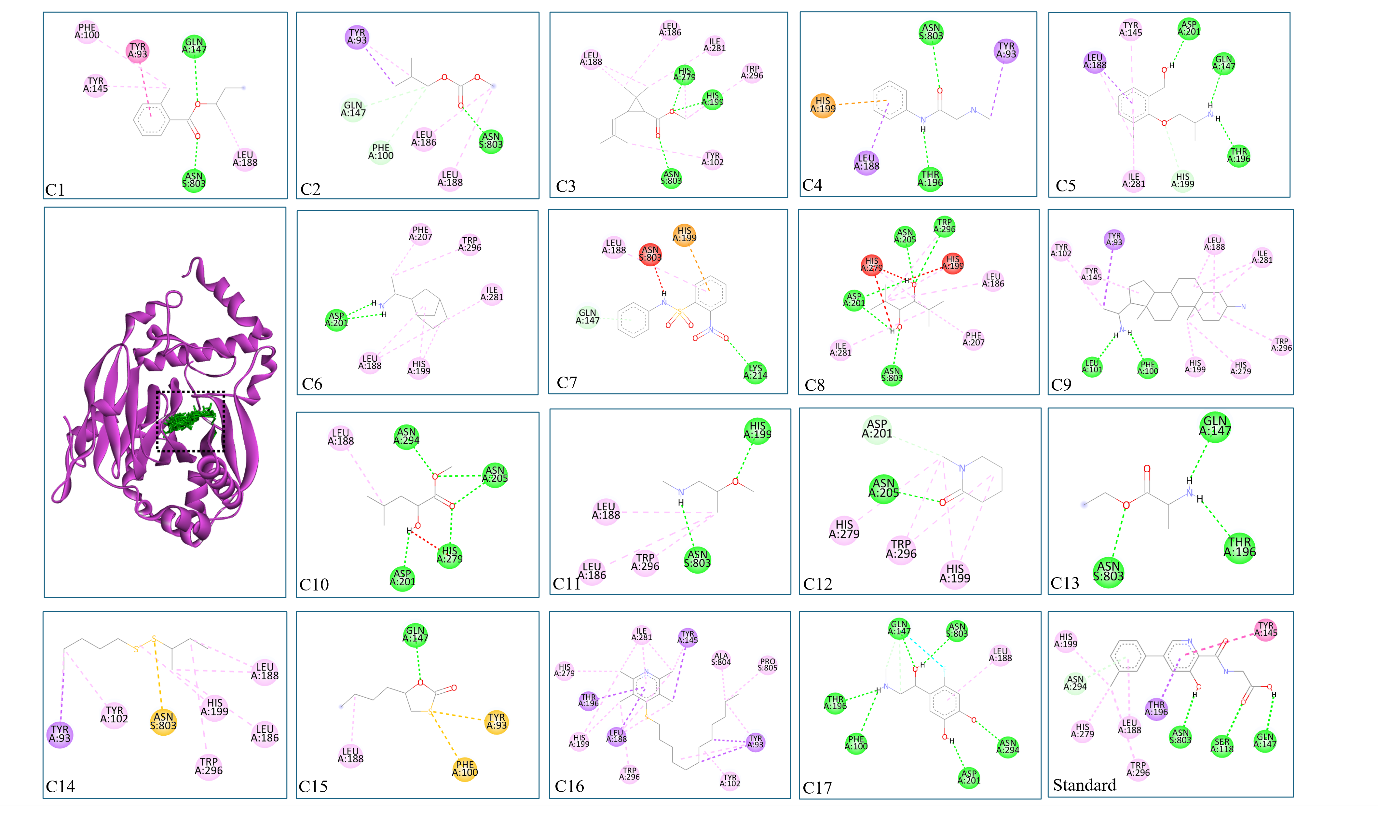
4 HIF1A.

Figure 4 2D visualization of HIF1A protein with lead compounds and reference.

Figure
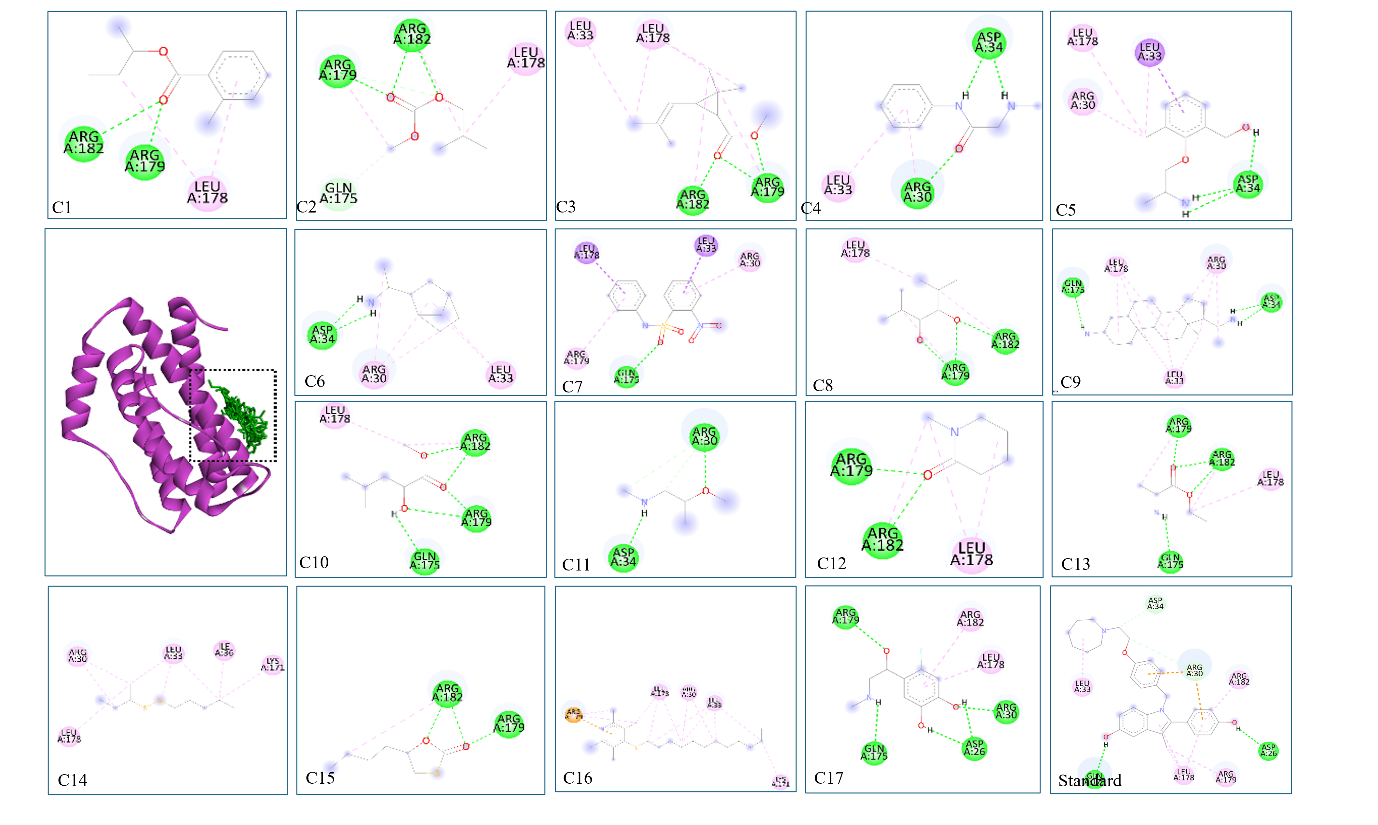
5 IL-6

Figure 5 2D visualization of IL-6 protein with lead compounds and reference.


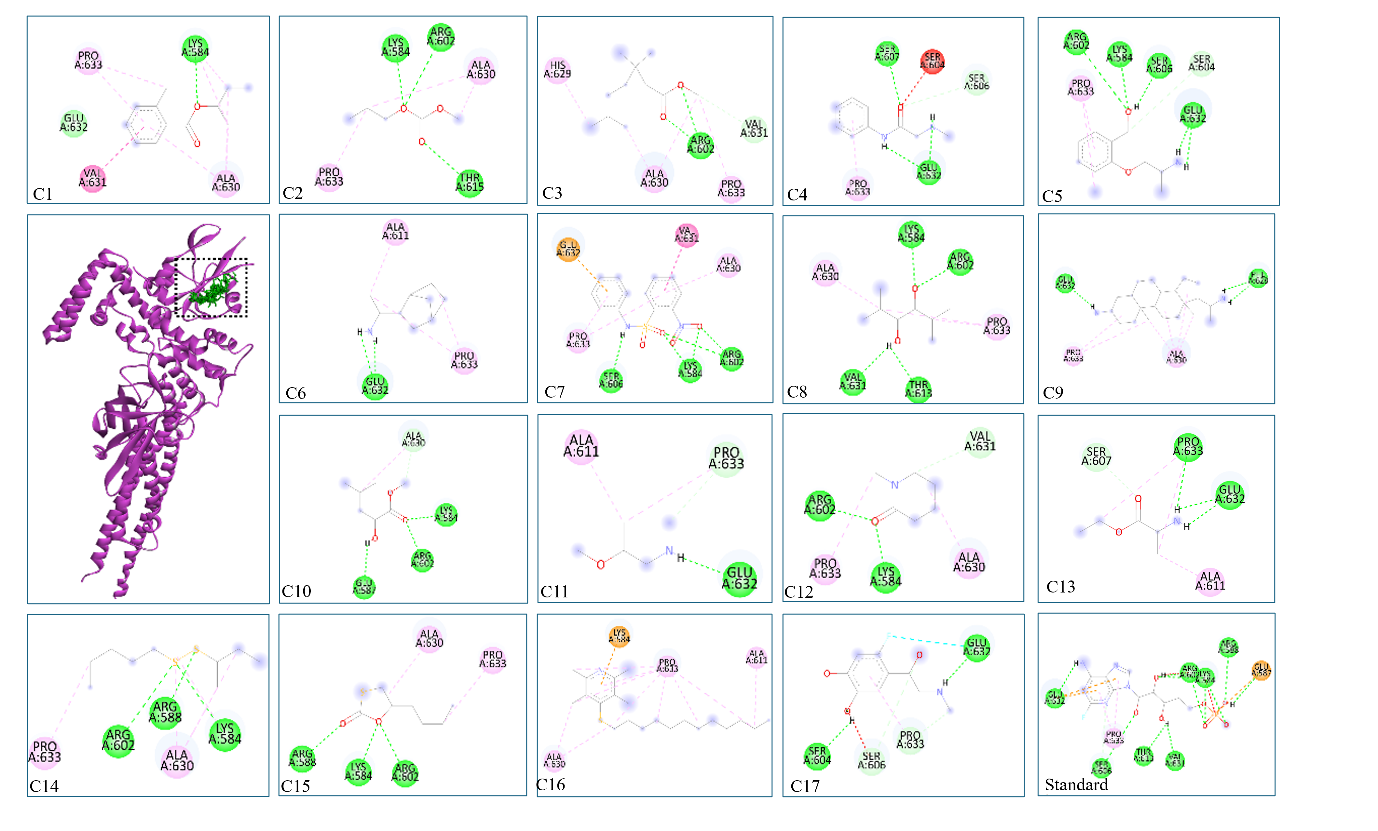
Figure 6 STAT1

Figure 6 2D visualization of STAT1 protein with lead compounds and reference.

Figure
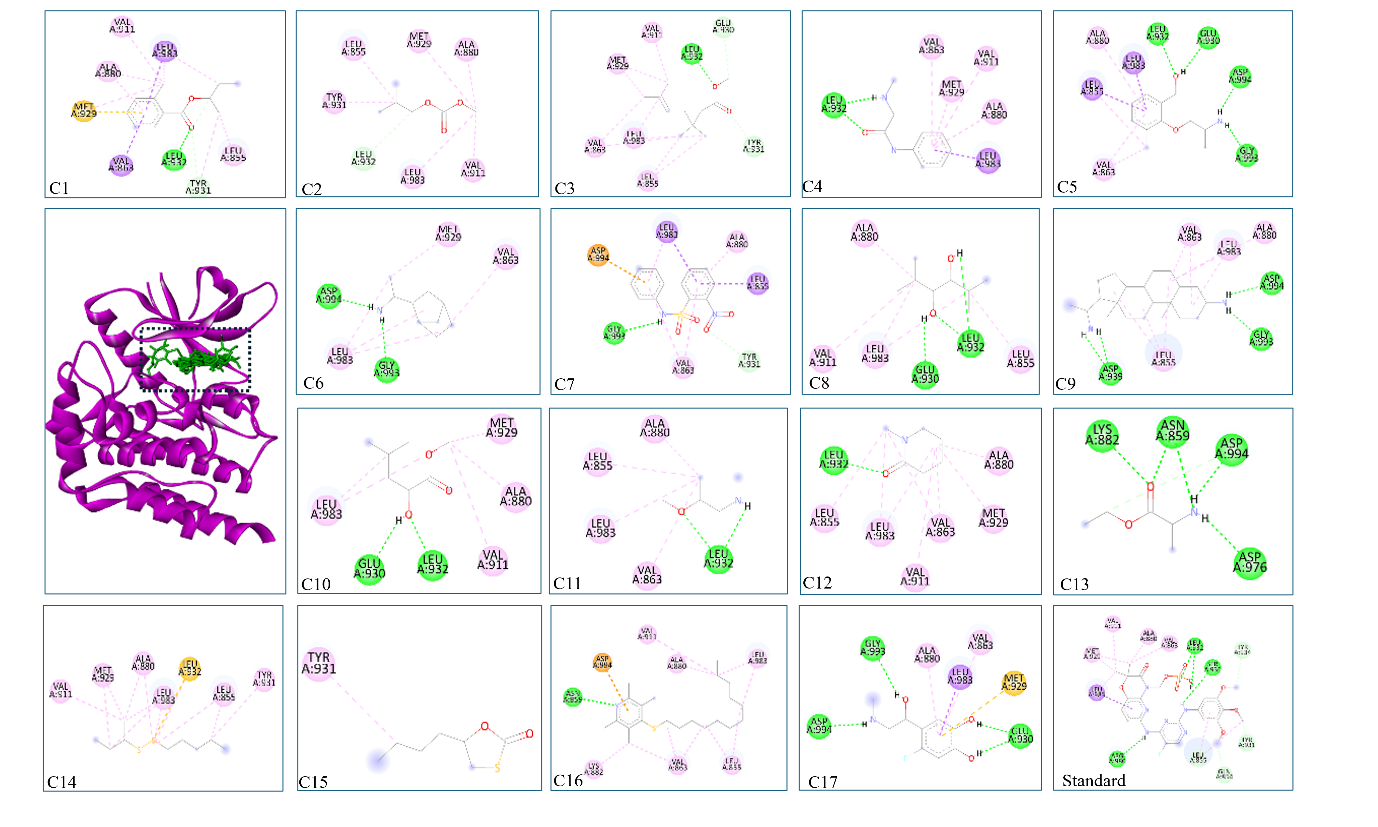
7 JAK2

Figure 7 2D visualization of JAK2 protein with lead compounds and reference.


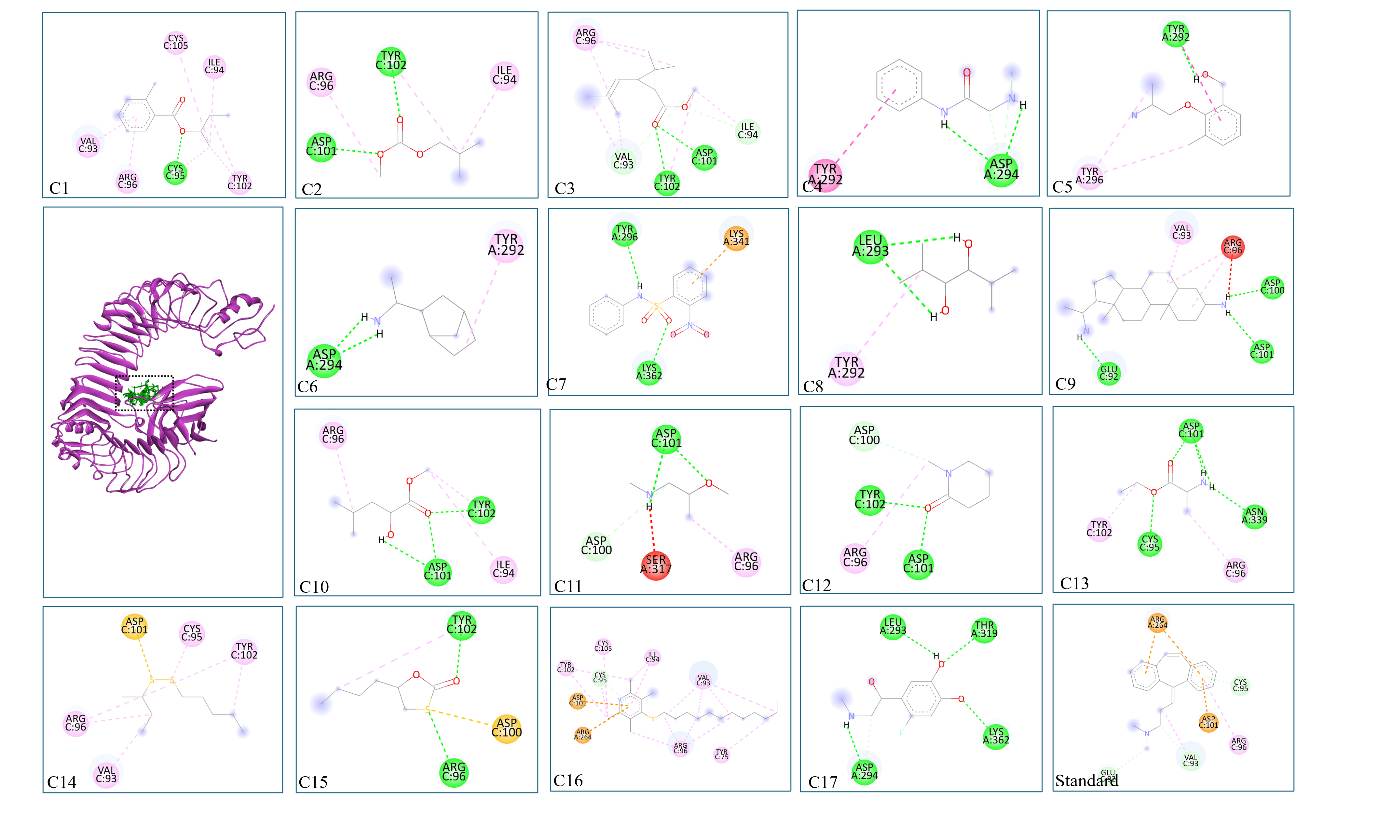

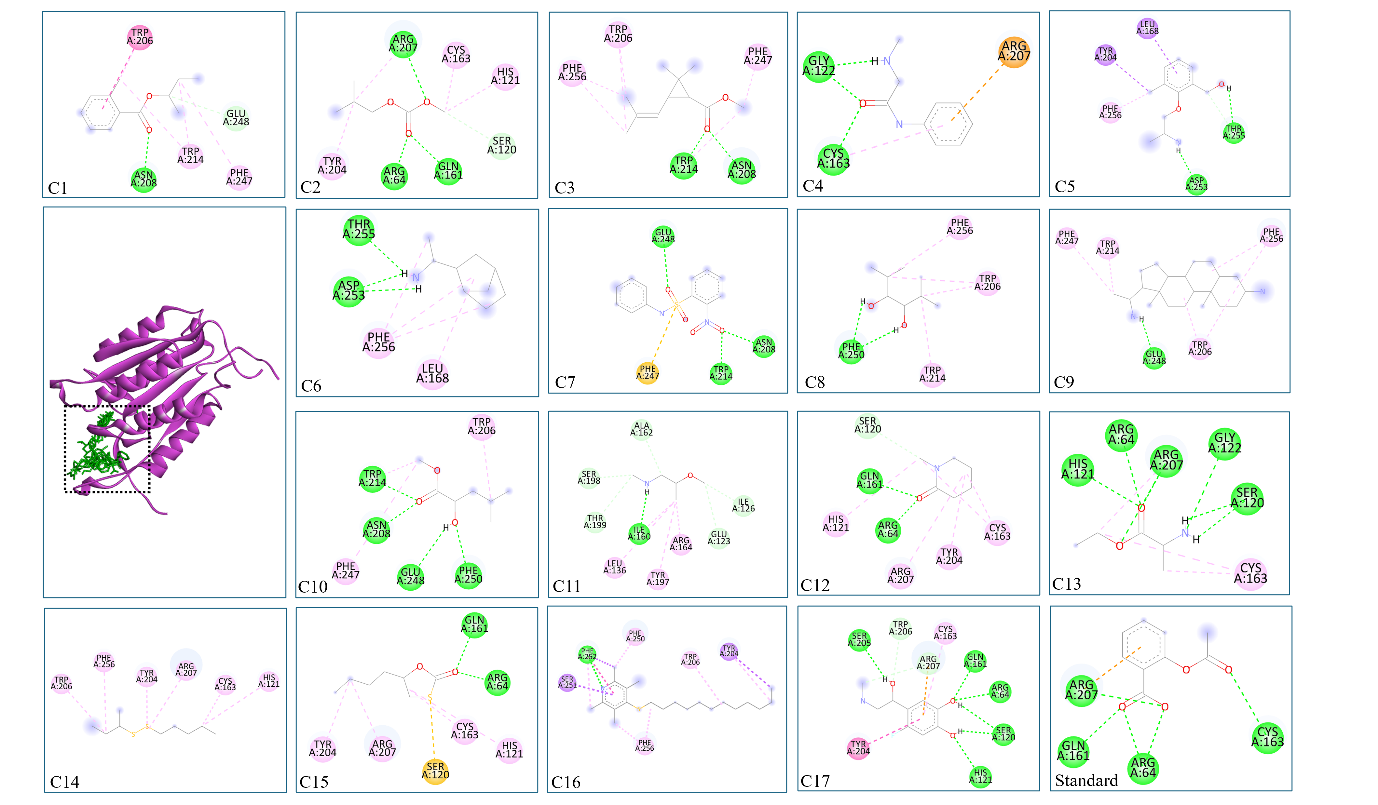
Figure 8 TLR4.

Figure 8 2D visualization of TRL4 protein with lead compounds and reference.

Figure 9 CASP3

Figure 9 2D visualization of CASP3 protein with lead compounds and reference.

Figure
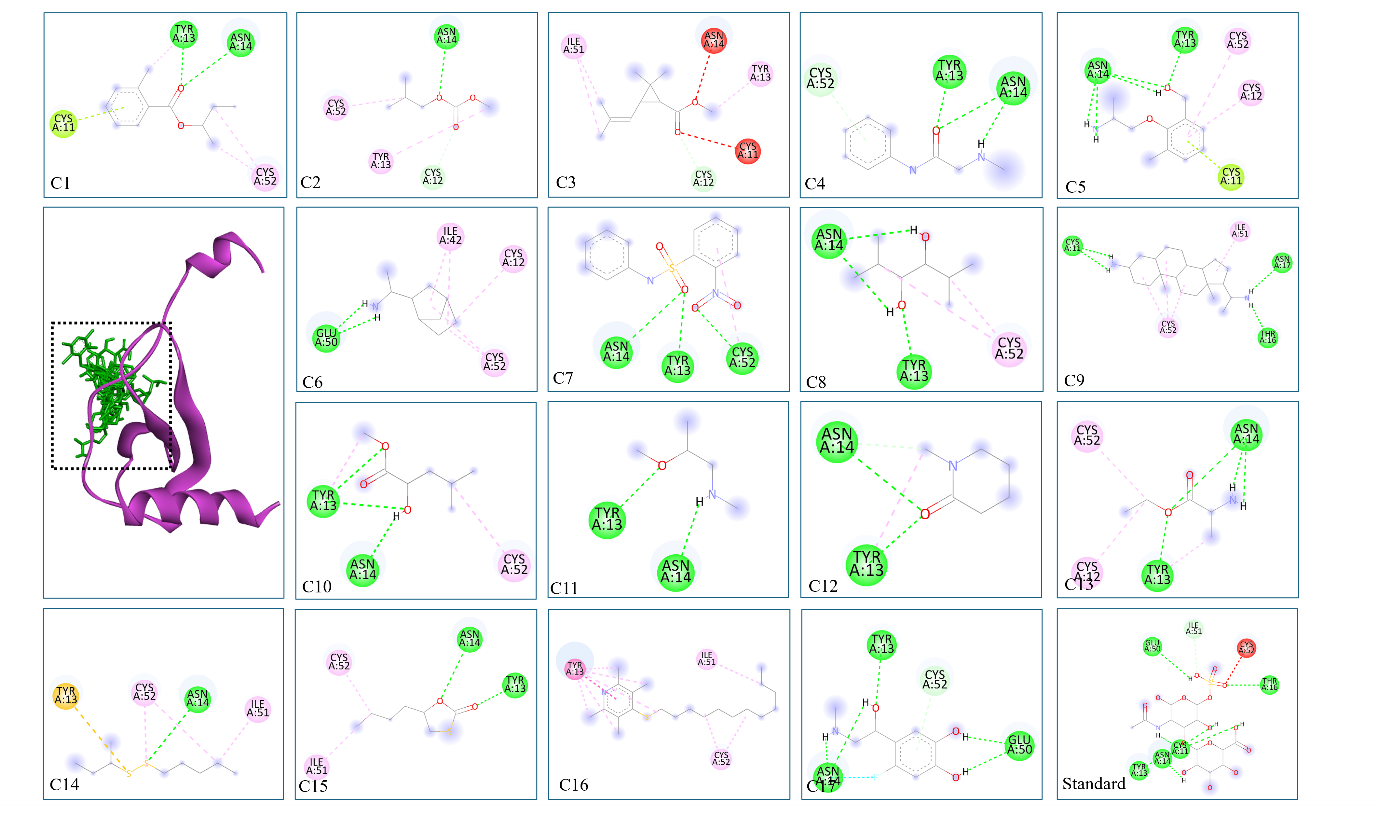
10 CCL2.

Figure 10 2D visualization of CCL2 protein with lead compounds and reference.


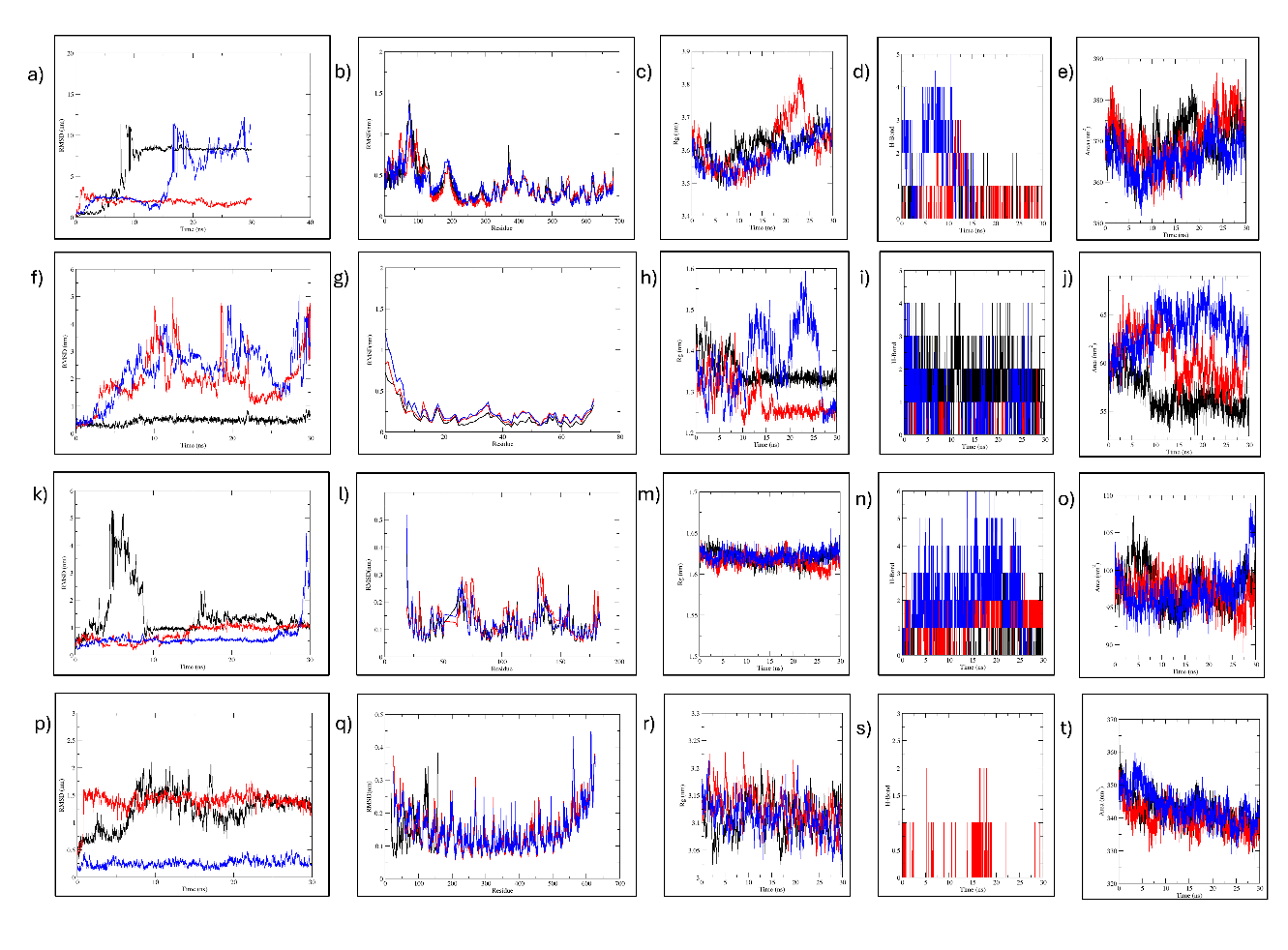
Figure 11.

Figure 11 MD simulation analysis of STAT1, CCL2, IL-6 and TLR4. a-e) STAT1-C7, C9 and R analysis (a)RMSD, (b)RMSF, (c)RoG, (d)H-bond, (e) SASA analysis. f-j) CCL2-C7, C9 and R analysis (f)RMSD, (g)RMSF, (h)RoG, (i)H-bond, (j) SASA analysis. k-o) IL-6-C7, C9 and R analysis (f)RMSD, (g)RMSF, (h)RoG, (i)H-bond, (j) SASA analysis. p-t) TLR4-C7, C9 and R analysis (p)RMSD, (q)RMSF, (r)RoG, (s)H-bond, (t) SASA analysis. Black represents C7, Red represent C9, and Blue represent the reference.

**Table 1 Protein structures PDB ID, grid coordinates and the binding sites**

| **Protein** | **PDB ID** | **Resolution** | **Grid coordinates** | | **Binding Sites** | | **Rreferences** |
| --- | --- | --- | --- | --- | --- | --- | --- |
|  |  |  |  |  | Binding Pocket | Amino acid residues |  |
| IL-6 | 1ALU | 1.90 Å | X | -4.295 | D-helix | Arg30, Arg175, Arg178, Arg179, Arg182 | [21], [22] |
|  |  |  | Y | -11.711 |  |  |  |
|  |  |  | Z | 3.142 |  |  |  |
| STAT1 | 1YVL | 3.00 Å | X | -26.126 | SH2 domain | Lys584, Arg602, Ser604, Glu605, Ser606, Glu618, His629, Ala630, Val631, Glu632, Pro633, Tyr634 | [23], [24] |
|  |  |  | Y | 3.398 |  |  |  |
|  |  |  | Z | 116.141 |  |  |  |
| PTGS2 | 5F1A | 2.38 Å | X | 44.316 | Cyclooxygenase active site of COX-2 | His90, Arg120, Tyr355, Tyr385, Arg513, E524, Ser530, L531 | [25], [26] |
|  |  |  | Y | 22.717 |  |  |  |
|  |  |  | Z | 242.007 |  |  |  |
| MMP9 | 4XCT | 1.30 Å | X | 18.3 | MMP9 S1’ inhibitor-binding pocket | Ala189, His190, Gly186, Leu222, His230, His236, Leu243, Pro246, Tyr248 | [27] |
|  |  |  | Y | -13.463 |  |  |  |
|  |  |  | Z | 19.084 |  |  |  |
| JAK2 | 3TJC | 2.40 Å | X | 117.86 | ATP binding site of kinase domain of JAK2 | Leu932, Glu930, Pro933, Asp994, Tyr931, Ala880, Leu855, Leu983, Val863 | [28], [29] |
|  |  |  | Y | 68.224 |  |  |  |
|  |  |  | Z | 8.524 |  |  |  |
| CCL2 | 1DOK | 1.85 Å | X | 10.023 | Receptor binding site. | Val9, Thr10, Cys11, Cys12, Thr13, Asn14, Ile42, Glu50, Ile51, Cys52 | [30], [31] |
|  |  |  | Y | 41.262 |  |  |  |
|  |  |  | Z | 39.243 |  |  |  |
| CASP3 | 1NMS | 1.70 Å | X | -8.162 | Catalytic domain (peptidase) | Arg64, Ser120, His121, Gly122, Gln161, Ala162, Cys163, Ser205, Trp206, Arg207, Trp204, Asn208, Ser209, Trp214, Ser249, Phe250, Phe256 | [32], [33] |
|  |  |  | Y | -4.446 |  |  |  |
|  |  |  | Z | 25.438 |  |  |  |
| HIF1A | 1H2M | 2.50 Å | X | 21.207 | Catalytic site | Tyr145, Leu188, Thr196, His199, Asn205, Phe207, Lys214, His279, Ile284, Asn294, Trp296 | [34], [35] |
|  |  |  | Y | 24.209 |  |  |  |
|  |  |  | Z | 27.922 |  |  |  |
| TLR4 | 3FXI | 3.10 Å | X | 23.733 | LPS Binding Pocket between TLR4 and MD2 | Val93, Asp101, Ser120, Phe121, Arg264, Tyr292, Leu293, Asp294, Tyr296, Ser317, Val318, Thr319, Asn339, Cys340, Lys341, Lys362 | [36], [37] |
|  |  |  | Y | -14.368 |  |  |  |
|  |  |  | Z | 17.393 |  |  |  |

**Table 2 IC-50 of DPPH, Albumin denaturation and proteinase assay**

| **Extract** | **DPPH** | **Albumin denaturation assay** | **Proteinase Assay** |
| --- | --- | --- | --- |
|  | **IC-_50_** | **IC-_50_** | **IC-_50_** |
| **AM** | 45.68 | 34.09 | 51.36 |
| **HA** | 102.77 | 64.52 | 78.25 |
| **Aq** | 287.34 | 146.6 | 105.03 |
| **E** | 112.22 | 96.50 | 100.95 |
| **Ascorbic Acid/ Aspirin** | 69.90 | 109.70 | 87.60 |

**Table 3 Degree of 17 lead compounds examined through cystoscape.**

| **Compound** | **Degree** | **MCC** | **MNC** | **Betweenness** | **Closeness** |
| --- | --- | --- | --- | --- | --- |
| **C9** | 60 | 60 | 1 | 3695.115 | 100.6667 |
| **C7** | 58 | 58 | 1 | 3627.807 | 99.33333 |
| **C10** | 57 | 57 | 1 | 2825.277 | 98.66667 |
| **C8** | 54 | 54 | 1 | 2124.077 | 96.66667 |
| **C6** | 54 | 54 | 1 | 2252.064 | 96.66667 |
| **C11** | 52 | 52 | 1 | 2356.328 | 95.33333 |
| **C3** | 49 | 49 | 1 | 2141.167 | 93.33333 |
| **C2** | 47 | 47 | 1 | 1444.783 | 92 |
| **C16** | 46 | 46 | 1 | 3045.789 | 91.33333 |
| **C13** | 45 | 45 | 1 | 2401.804 | 90.66667 |
| **C5** | 44 | 44 | 1 | 2526.12 | 90 |
| **C12** | 43 | 43 | 1 | 2084.097 | 89.33333 |
| **C17** | 42 | 42 | 1 | 2212.773 | 88.66667 |
| **C15** | 42 | 42 | 1 | 1164.699 | 88.66667 |
| **C14** | 39 | 39 | 1 | 1047.26 | 86.66667 |
| **C1** | 38 | 38 | 1 | 1837.181 | 86 |
| **C4** | 27 | 27 | 1 | 1157.657 | 78.66667 |

| **Protein**  **Table 4: Interactions and binding energies between putative active substances and their protein targets.** | **PDB ID** | **Compounds** | **Binding Energies** | **Inhibition Constant (Ki)** | **Hydrogen bonds** | **Hydrophobic interactions** | **Common residues with standards** |
| --- | --- | --- | --- | --- | --- | --- | --- |
| **STAT1** | 1YVL | C1 | -4.39 | 604.62 uM | Lys584, Glu632 | Val631, Pro633, Ala630 | Lys584, Pro633, |
|  |  | C2 | -3.57 | 2.40 mM | Lys584, Arg602, Thr615 | Ala630, Pro633 | Lys584, Arg602, Pro633 |
|  |  | C3 | -4.14 | 925.09 uM | Arg602, Val631 | His629, Ala630, Pro633 | Arg602, Val631, Pro633 |
|  |  | C4 | -4.64 | 499.52 uM | Ser607, Glu632, Ser606 | Pro633 | Glu632, Ser606, Pro633 |
|  |  | C5 | -5.25 | 141.66 uM | Arg602, Lys584, Ser606, Glu632, Ser604 | Pro633 | Arg602, Lys584, Ser606, Glu632, Pro633 |
|  |  | C6 | -4.87 | 267.81 uM | Glu632 | Ala611, Pro633 | Glu632, Pro633 |
|  |  | C7 | -7.53 | 3.02 uM | Ser606, Lys584, Arg602 | Pro633, Glu632, Val631, Ala630 | Ser606, Lys584, Arg602, Pro633, Glu632, Val631 |
|  |  | C8 | -3.58 | 2.37 mM | Lys584, Arg602, Val631, Thr613 | Ala630, Pro633 | Lys584, Arg602, Val631, Thr613, Pro633 |
|  |  | C9 | -5.5 | 92.83 uM | Glu632, Phe628 | Pro633, Ala630 | Glu632, Pro633 |
|  |  | C10 | -4.64 | 2.59 mM | Ala630, Lys584, Arg602, Glu587 | Ala630 | Lys584, Arg602, Glu587, Ala630 |
|  |  | C11 | -3.01 | 6.25 mM | Glu632, Pro633 | Ala611, Pro633 | Glu632, Pro633, Pro633 |
|  |  | C12 | -3.69 | 1.96 mM | Arg602, Lys584, Val631 | Pro633, Ala630 | Arg602, Lys584, Val631, Pro633 |
|  |  | C13 | -3.9 | 1.38 mM | Pro633, Glu632, Ser607 | Ala611, Pro633 | Pro633, Glu632, Pro633 |
|  |  | C14 | -2.99 | 14.17 mM | Arg602, Arg588, Lys584 | Pro633, Ala630, Lys584 | Arg602, Arg588, Lys584, Pro633, Lys584 |
|  |  | C15 | -4.24 | 784.23 uM | Arg588, Lys584, Arg602 | Ala630, Pro633 | Pro633 |
|  |  | C16 | -3.2 | 4.53 mM | - | Ala630,Lys584, Pro633, Ala611 | Lys584, Pro633 |
|  |  | C17 | -4.11 | 975.15 uM | Ser604, Glu632, Ser606, Pro633 | Pro633, Glu632 | Glu632, Ser606, Pro633 |
|  |  | Fluradibine phosphate | -5.41 | 108.65uM | Glu632,Ser606,Thr613,Val631,Arg602,Lys584,Arg588, Glu587 | Glu632, Pro633, Glu587 | - |
| **MMP9** | 4XCT | C1 | -8.71 | 411.05 nM | Arg249 | Arg249,Leu222,Tyr248, Val223, His226 | Ard249, Leu222, Tyr248, His 226 |
|  |  | C2 | -5.48 | 95.39 uM | Ala242, Arg249 | Arg249, Tyr248, His226 | Ala242, Arg249, Tyr248, His226 |
|  |  | C3 | -7.99 | 1.39 uM | Pro240 | Leu188, Val223, Tyr248, Leu222, Leu243, Tyr245, Arg249, His226 | Tyr248, Leu222, Leu243, Tyr245, Arg249, His226 |
|  |  | C4 | -9.97 | 49.52 nM | Pro240, Ala242, Tyr248, Met247, Arg249, Tyr245, Leu243 | Val223, Tyr248, His226 | Ala242, Tyr248, Arg249, Tyr245, Leu243, His226 |
|  |  | C5 | -8.49 | 594.49 nM | Arg249, Met247, Leu243, Tyr245 | Leu243, Val223, His226 | Arg249, Leu243, Tyr245, His226 |
|  |  | C6 | -8.03 | 1.30 uM | Tyr245, Ala242, Met247 | Arg249, Tyr248, His 226, Leu243, Leu222 | Tyr245, Ala242, Arg249, Tyr248, His226, Leu243 |
|  |  | C7 | -9.98 | 48.31 nM | Tyr245, His226  Arg249 | Val223 | Tyr245, His226, Arg249 |
|  |  | C8 | -7.03 | 6.99 uM | Arg249, Met247 | His226, Tyr248 | Arg249, His226, Tyr248 |
|  |  | C9 | -15.07 | 8.99 pM | Arg249, Ala189 | His226, Val223, Leu188, Tyr248, Pro255, Leu222, Leu243 | Arg249, His226, Leu243, Leu222, Tyr248 |
|  |  | C10 | -8.46 | 633.10 nM | Ala242,Arg249, Pro240, Met247 | His226, Leu243, Arg249 | Ala242, Arg249, Leu243, Arg249, His226 |
|  |  | C11 | -5.2 | 155.14 uM | Arg249, Met247, His226, Tyr245 | Leu243, His226 | Arg249, His226, Tyr245, Leu243, His226 |
|  |  | C12 | -5.82 | 54.50 uM | Arg249 | His226, Tyr248 | Arg249, His226, Tyr248 |
|  |  | C13 | -6.17 | 30.01 uM | Arg249, Tyr245, Met247 | His226, Arg249 | Arg249, Tyr245, His226, Arg249 |
|  |  | C14 | -6.75 | 11.20 uM | Arg249 | His226, Leu222, Tyr248 | Arg249, His226, Leu222, Tyr248 |
|  |  | C15 | -8.36 | 750.56 nM | Arg249 | Arg249 | Arg249 |
|  |  | C16 | -10.03 | 44.68 nM | Leu187 | His226, His236, Leu188, Leu243, Val223, Tyr248, Leu222, Arg249 | His226, Leu243, Tyr248, Leu222, Arg249 |
|  |  | C17 | -6.86 | 9.35 uM | Pro240, Ala242, Tyr245, Leu243 | His226, Leu222 | Ala242, Tyr245, Leu243, His226, Leu222 |
|  |  | Minocycline | -9.36 | 138.06nM | Gly186, Leu243, Ala242, Tyr245 | Leu222, Tyr248, Arg249, His226 | Gly186, Leu243, Ala242, Tyr245, Leu222, Tyr248, Arg249, His226 |
| **IL-6** | 1ALU | C1 | -4.65 | 422.26 uM | Arg179, Arg182 | Leu178 | Arg179, Arg182, Leu178 |
|  |  | C2 | -3.68 | 1.99 mM | Arg179, Gln175, Arg182 | Leu178, Arg182, Arg179 | Arg179, Gln175, Arg182, Arg179 |
|  |  | C3 | -4.83 | 289.20 uM | Arg179, Arg182 | Leu33, LEU178, Arg179, Arg182 | Arg179, Arg182, Leu33, Leu178, Arg179, Arg182 |
|  |  | C4 | -5.39 | 253.50 uM | Arg30, Asp34 | Arg30, Leu33 | Arg30, Leu33, Asp34 |
|  |  | C5 | -4.81 | 300.04 uM | Asp34 | Leu33, Leu178, Arg30 | Asp34, Leu33, Leu178, Arg30 |
|  |  | C6 | -5.1 | 182.49 uM | Asp34 | Arg30, Leu33 | Asp34, Arg30, Leu33 |
|  |  | C7 | -7.07 | 6.60 uM | Gln175, Arg179, Arg182 | Leu178, Arg179 | Gln175, Arg179, Arg182, Leu178, Arg179 |
|  |  | C8 | -3.63 | 2.19 mM | Arg179, Arg182 | Leu178, Arg179, Arg182 | Arg179, Arg182, Leu178 |
|  |  | C9 | -6.33 | 22.86 uM | Gln175, Asp34 | Leu178, Arg30, Leu33 | Gln175, Asp34, Leu178, Arg30, Leu33 |
|  |  | C10 | -5.02 | 1.76 mM | Gln175, Arg179, Arg182 | Leu178, Arg182 | Gln175, Arg179, Leu178, Arg182 |
|  |  | C11 | -3.78 | 8.06 mM | Asp34, Asp30 | - | Asp34, Asp30 |
|  |  | C12 | -3.73 | 1.84 mM | Arg179, Arg182 | Leu178, Arg182 | Arg179, Arg182, Leu178, Arg182 |
|  |  | C13 | -4.74 | 333.67 uM | Gln175, Arg179, Arg182 | Arg179, Arg182, Leu178 | Gln175, Arg179, Leu178 |
|  |  | C14 | -3.03 | 5.99 mM |  | Arg30, Leu178, Leu33, Ile36, Lys171 | Arg30, Leu178, Leu33 |
|  |  | C15 | -4.58 | 439.46 uM | Arg182, Arg179 | Arg182 | Arg182, Arg179 |
|  |  | C16 | -4.09 | 998.96 uM |  | Arg179, Lys171, Leu178, Arg30, Leu33 | Arg179, Leu178, Arg30, Leu33 |
|  |  | C17 | -4.5 | 505.33 uM | Arg179, Gln175, Asp26, Arg30 | Arg182, Leu178 | Arg179, Gln175, Asp26, Arg30, Arg182, Leu178 |
|  |  | Bazedoxifene | -4.65 | 422.26 uM | Gln175, Asp26, Arg30, Asp34 | Leu33, Arg182, Leu178, Arg179 | - |
| **PTGS2** | 5F1A | C1 | -5.94 | 44.44 uM | Ala527 | Leu531, Val349, Gly526, Leu352, Trp387, Phe381, Tyr285, Met522 | Ala527, Leu531, Val349, Gly526, Leu352, Met522 |
|  |  | C2 | -4.08 | 1.03 mM | Trp387, Tyr385 | His207, Leu390 | Tyr385 |
|  |  | C3 | -6.38 | 21.09 uM | Val523, Ala527 | Val349, Leu352, Phe381, Leu384, Tyr385, Phe518, Trp387, Met522, Tyr348, Ala527, Val523 | Val523, Ala527, Val349, Leu352, Tyr385, Ala527, Val523 |
|  |  | C4 | -7.85 | 86.04 uM | -- | Tyr385, Ser530 | Tyr385, Ser530 |
|  |  | C5 | -5.38 | 113.97 uM | Ala202, Thr206, Tyr385, Trp387 | Leu390, His388, His386, His207, Phe210, Trp387, Ala202 | Tyr385 |
|  |  | C6 | -6.04 | 37.39 uM | Ser530, Tyr385 | Val349, Leu352, Val523, Trp387, Ala527 | Ser530, Tyr385, Val349, Leu352, Val523, Ala527 |
|  |  | C7 | -6.8 | 10.28 uM | Ala527, Ser530 | Gly526, Leu352, Met522, Val523, Phe518, Leu531, Val349, Ala527 | Ala527, Ser530, Gly526, Leu352, Met522, Val523, Leu531, Val349, Ala527 |
|  |  | C8 | -4.54 | 473.34 uM | Tyr385 | Val349, Leu352, Tyr348 | Tyr385, Val349, Leu352 |
|  |  | C9 | -8.76 | 379.05 nM | Val582, Asp584, Thr94 | Ala516, His90 | - |
|  |  | C10 | -6.13 | 368.39 uM | Tyr385, Thr206 | Tyr385, His207, Leu391, Leu390 | Tyr385 |
|  |  | C11 | -3.08 | 5.49 mM | Tyr385, Met522 | Trp387, Leu384 | Tyr385, Met522 |
|  |  | C12 | -4.83 | 287.68 uM | Tyr385, Trp387 | Leu390, Ala199, Leu391, Ala202 | Tyr385 |
|  |  | C13 | -3.63 | 2.18mM | Tyr385, Ser530 | Trp387, Leu384, Met522, Leu352, Val349 | Tyr385, Ser530, Met522, Leu352, Val349 |
|  |  | C14 | -5.03 | 206.46 uM | - | Phe381,Leu352, Tyr385, Phe205, Tyr348, Val344, Ala527, Met522, Phe518, Trp387 | Leu352, Tyr385, Tyr385, Ala527 |
|  |  | C15 | -6.6 | 14.57 uM | Trp387 | Phe210, His207, Tyr385, Ala202, Trp387 | Tyr385 |
|  |  | C16 | -7.27 | 4.69uM | - | Tyr355, Leu531, Ala527, Val523, Leu352, Val349, Phe518, Tyr348, Val344, Phe205, Leu534, Phe209, Val228, Phe381 | Leu352, Tyr355, Val523, Leu352, Ala527, Val349, Leu531 |
|  |  | C17 | -4.47 | 527.64 uM | Tyr385, Met522, Ser530 | Gly526, Leu352, Trp387 | Tyr385, Met522, Ser530, Gly526, Leu352 |
|  |  | Niflumic acid | -5.57 | 82.0uM | Ser353, Arg120, Tyr385, Ser530 | Tyr355, Val523, Met522, Leu352, Gly526, Ala527, Val349, Leu531 | - |
| **JAK2** | 3TJC | C1 | -5.89 | 48.30 uM | Leu932, Tyr931 | Leu855, Val863, Tyr931, Val911, Ala880, Met929, Leu983 | Leu932, Tyr931, Leu855, Val863, Val911, Ala880, Met929, Leu983 |
|  |  | C2 | -3.97 | 1.24 mM | Leu932 | Tyr931, Leu855, Met929, Ala880, Val911, Leu983 | Leu932, Tyr931, Leu855,Met929, Val911, Leu983 |
|  |  | C3 | -5.54 | 87.30 uM | Leu932, Tyr931, Glu930 | Val911, Met929, Val863, Leu983, Leu855 | Leu932, Tyr931, Val911, Met929, Val911, Val863, Leu983, Leu855 |
|  |  | C4 | -6.61 | 14.38 uM | Leu932 | Val863, Met929, Val911, Ala880, Leu983 | Leu932, Val863, Met929, Val911,Ala880, Leu983 |
|  |  | C5 | -6.64 | 13.59 uM | Leu932, Glu930, Asp994, Gly993 | Ala880, Leu983, Leu855, Val863 | Leu932, Ala880, Leu983, Leu855, Val863 |
|  |  | C6 | -5.34 | 121.06 uM | Asp994, Gly993 | Met929, Val863, Leu983 | Met929, Val863, Leu983 |
|  |  | C7 | -6.89 | 8.87 uM | Gly993, Tyr931 | Asp994, Leu983, Ala880, Leu855, Val863 | Tyr931, Leu983, Ala880, Leu855, Val863 |
|  |  | C8 | -4.91 | 250.26 uM | Glu930, Leu932 | Leu855, Leu983, Val911, Ala880 | Leu932, Leu855, Leu983, Val911, Ala880 |
|  |  | C9 | -10.12 | 38.20 nM | Asp939, Asp994, Gly993 | Val863, Ala880, Leu983, Leu855 | Val863, Ala880, Leu983, Leu855 |
|  |  | C10 | -4 | 1.17 mM | Glu930, Leu932 | Leu983, Met929, Ala880, Val911 | Leu932, Leu983, Met929, Ala880, Val911 |
|  |  | C11 | -4.72 | 345.18 uM | Leu932 | Ala880, Leu855, Leu983, Val863 | Leu932, Ala880, Leu855, Leu983, Val863 |
|  |  | C12 | -4.33 | 671.83 uM | Leu932 | Ala880, Met929, Val863, Val911, Leu983, Leu855 | Leu932, Ala880, Met929, Val863, Val911, Leu983, L855 |
|  |  | C13 | -4.56 | 453.94 uM | Lys882, Asn859, Asp994, Asp976 | - | - |
|  |  | C14 | -4.39 | 609.90 uM | - | Val911, Met929, Ala880, Leu983, Leu932, Leu855, Tyr931 | Val911, Met929, Ala880, Leu983, Leu932, Leu855, Tyr931 |
|  |  | C15 | -6.03 | 37.97 uM | - | Tyr931 | Tyr931 |
|  |  | C16 | -6.99 | 7.53 uM | Asn859 | Asp994, Val911, Ala880, Leu983, Leu855, Val863, Lys882 | Val911, Ala880, Leu855, Val863 |
|  |  | C17 | -5.58 | 81.19 uM | Asp994, Gly993, Glu930 | Ala880, Val863, Leu983, Met929 | Ala880, Val863, Leu983, Met929 |
|  |  | Fostamatinib | -6.84 | 9.73uM | Leu932, Ser936, Tyr934, Tyr931, Gln854, Leu855, Arg980 | Leu983,Met929, Val911, Ala880, Val863, Leu855 | - |
| **CCL2** | 1DOK | C1 | -4.99 | 218.37 uM | Tyr13, Asn14 | Cys52, Cys11 | Tyr13, Asn14, Cys11G |
|  |  | C2 | -3.49 | 2.75 mM | Asn14, Cys12 | Tyr13, Cys52 | Asn14, Tyr13 |
|  |  | C3 | -4.95 | 235.58 uM | Cys12 | Ile51, Tyr13 | Ile51, Tyr13 |
|  |  | C4 | -5.78 | 57.92 uM | Cys52, Cys12, Tyr13, Asn14 | - | Tyr13, Asn14 |
|  |  | C5 | -4.06 | 1.06 mM | Asn14, Tyr13 | Cys11, Cys12, Cys52 | Asn14, Tyr13, Cys11 |
|  |  | C6 | -4.19 | 846.85 uM | Glu50 | Ile42, Cys12, Cys52 | Glu50 |
|  |  | C7 | -5.87 | 50.01 uM | Asn14, Tyr13, Cys52 | Cys52 | Tyr13, Asn14 |
|  |  | C8 | -4.14 | 929.84 uM | Asn14, Tyr13 | Cys52 | Asn14, Tyr13 |
|  |  | C9 | -6.78 | 10.64 uM | Cys11, Asn17, Thr16 | Cys52, Ile51 | Thr16, Ile51 |
|  |  | C10 | -3.75 | 1.79 mM | Tyr13, Asn14 | Cys52, Tyr13 | Tyr13, Asn14 |
|  |  | C11 | -4.05 | 1.07 mM | Tyr13, Asn14 |  | Tyr13, Asn14 |
|  |  | C12 | -4.46 | 534.09 uM | Asn14, Tyr13 | Tyr13 | Asn14, Tyr13 |
|  |  | C13 | -3.15 | 4.94 mM | Asn14, Tyr13 | Cys52, Cys12, Tyr13 | Asn14, Tyr13 |
|  |  | C14 | -3.76 | 1.75 mM | Asn14 | Ile51, Cys52, Tyr13 | Asn14, Ile51, Tyr13 |
|  |  | C15 | -4.31 | 689.79 uM | Asn14, Tyr13 | Cys52, Ile51 | Asn14, Tyr13, Ile51 |
|  |  | C16 | -5.5 | 92.57 uM |  | Tyr13, Ile51, Cys52 | Tyr13, Ile51 |
|  |  | C17 | -4.44 | 558.16 uM | Asn14, Tyr13, Glu50, Cys52 | Asn14 | Tyr13, Asn14, Glu50 |
|  |  | Chondroitin 4-sulfate | -5.7 | 66.65uM | Tyr13, Asn14, Cys11, Thr16, Glu50, Ile51 | - | - |
| **CASP3** | 1NMS | C1 | -4.66 | 382.89 uM | Asn208, Glu248 | Trp206, Trp214, Phe247 | - |
|  |  | C2 | -4.55 | 459.27 uM | Arg207, Arg64, Gln161, Ser120 | Cys163, His121, Tyr204 | Arg64, Gln161, Cys163, Arg207 |
|  |  | C3 | -4.61 | 416.36 uM | Trp214, Asn208 | Phe247, Trp206, Phe256, Trp214 | - |
|  |  | C4 | -4.43 | 566.17 uM | Gly122, Cys163 | Arg207, Cys163 | Cys163 |
|  |  | C5 | -4.1 | 983.72 uM | Thr255, Asp253 | Leu168, Tyr204, Phe256 | - |
|  |  | C6 | -4.46 | 535.01 uM | Thr255, Asp253 | Phe256, Leu168 | - |
|  |  | C7 | -5.21 | 151.91 uM | Glu248, Asn208, Trp214 | Phe247 | - |
|  |  | C8 | -3.95 | 1.28 mM | Phe250 | Phe256, Trp206, Trp214 | - |
|  |  | C9 | -6.56 | 15.41 uM | Glu248 | Trp206, Phe256, TRp214, Phe247 | - |
|  |  | C10 | -3.32 | 3.66 mM | Trp214, Asn208, Glu248, Phe250 | Phe247, Trp206 | - |
|  |  | C11 | -3.07 | 5.65 mM | Ala162, Ser198, Thr199, Ile160, Ile126, Glu123 | Leu136, Tyr197, Arg164 | - |
|  |  | C12 | -4.74 | 333.10 uM | Gln161, Arg64, Ser120 | His121, Arg207, Tyr204, Cys163 | Arg64, Gln161, Cys163, Arg207 |
|  |  | C13 | -5.44 | 102.05 uM | His121, Arg64, Arg207, Gly122, Ser120 | Cys163, Arg207 | Arg64, Cys163, Arg207 |
|  |  | C14 | -3.79 | 1.68 mM | Trp206, Phe256, Tyr204, Arg207, Cys163, His121 | - | Cys163, Arg207 |
|  |  | C15 | -5.72 | 63.65 uM | Gln161, Arg64 | Cys163, His121, Ser120, Arg207, Tyr204 | Arg64, Gln161, Cys163, Arg207 |
|  |  | C16 | -4.55 | 464.95 uM | Phe252 | Phe250, Ser251, Phe256, Trp206, Tyr204 | - |
|  |  | C17 | -4.54 | 467.85 uM | Ser205, Trp206, Arg207, Gln161, Arg64, Ser120, His121 | Tyr204, Cys163 | Arg64, Gln161, Cys163, Arg207 |
|  |  | Aspirin | -5.82 | 53.75uM | Arg64, Gln161, Cys163, Arg207 | Arg207 | Arg64, Gln161, Cys163, Arg207 |
| **TLR4** | 3FXI | C1 | -4.51 | 494.35 uM | Cys95 | Val93, Arg96, Tyr102, Ile94, Cys105 | Cys95, Val93, Arg96 |
|  |  | C2 | -4.27 | 742.24 uM | Tyr102, Asp101 | Ile94, Arg96, Tyr102 | Asp101, Arg96 |
|  |  | C3 | -5.04 | 203.81 uM | Val93, Tyr102, Asp101, Ile94 | Arg96, Val93, Ile94, Tyr102 | Val93, Asp101, Arg96, Val93 |
|  |  | C4 | -5.18 | 159.77 uM | Asp294 | Tyr292 | - |
|  |  | C5 | -4.69 | 362.12 uM | Tyr292 | Tyr296, Tyr292 | - |
|  |  | C6 | -5.48 | 96.45 uM | Asp294 | Tyr292 | - |
|  |  | C7 | -5.93 | 44.91 uM | Tyr296, Lys362 | Lys341 | - |
|  |  | C8 | -3.93 | 1.32 mM | Leu293 | Tyr292 | - |
|  |  | C9 | -7.8 | 1.92 uM | Glu92, Asp101, Asp100 | Val93, Arg96 | Glu92, Asp101, Val93, Arg96 |
|  |  | C10 | -4.34 | 658.73 uM | Tyr102, Asp101 | Ile94, Arg96, Tyr102 | Asp101, Arg96 |
|  |  | C11 | -4 | 1.17mM | Ile160, Ala162, Ser198,Thr199, Glu123, Ile126 | Leu136, Tyr197, Arg164 | - |
|  |  | C12 | -4.41 | 586.91uM | Gln161, Arg64, Ser120 | His121, Arg207, Tyr204, Cys163 | Gln161, Arg64, |
|  |  | C13 | -4.44 | 556.21 uM | Asp101, Asn339, Cys95 | Arg96, Tyr102 | Asp101, Cys95, Arg96 |
|  |  | C14 | -4.01 | 1.15mM | - | Trp206, Phe256, Tyr204, Arg207, Cys163, His121 | - |
|  |  | C15 | -5.09 | 187.32 uM | Tyr102, Arg96 | Asp100, Tyr102 | Arg96 |
|  |  | C16 | -4.7 | 359.38 | Phe252 | Phe252, Ser251, Phe250, Phe256, Trp206, Tyr204 | - |
|  |  | C17 | -5.18 | 160.03 uM | Leu293, Thr319, Lys362, Asp294 | - | - |
|  |  | Cyclobenzaprine | -5.67 | 70.12uM | Glu92, Val93,Cys95 | Arg264, Asp101, Arg96, Val93 | - |
| **HIF1A** | 1H2M | C1 | -6.29 | 24.57 uM | Gln147, Asn803 | Leu188, Tyr93, Tyr145, Phe100 | Leu188 |
|  |  | C2 | -4.83 | 290.14 uM | Asn803, Phe100, Gln147 | Tyr93, Leu186, Leu188 | Leu188 |
|  |  | C3 | -5.77 | 58.91 uM | His279, His199, Asn803 | Leu188, Leu186, Ile281, Trp296, Tyr102, His279, His199 | Leu188, His279, Ile281 |
|  |  | C4 | -6.99 | 7.49 uM | Asn803, Thr196 | His199, Leu188, Tyr93 | Leu188 |
|  |  | C5 | -6.16 | 30.53 uM | Asp201, Gln147, Thr196, His199 | Tyr145, Leu188, Ile281 | Leu188, Asp201, Ile281 |
|  |  | C6 | -5.94 | 44.38 uM | Asp201 | Phe207, Trp296, Ile281, His199, Leu188 | Leu188, Asp201, Ile281, |
|  |  | C7 | -6.76 | 11.00 uM | Lys214, Gln147 | Leu188, His199 | Leu188 |
|  |  | C8 | -4.76 | 322.96 uM | Asn803, Asp201, Asn205, Trp296 | Ile281, Phe207, Leu186 | Leu188, Asp201, Asn205 |
|  |  | C9 | -6.26 | 25.91 uM | Phe100, Leu101 | Tyr102, Tyr145, Tyr93, Leu188, Ile281, Trp296, His279, His199 | Leu188, His279 |
|  |  | C10 | -4.56 | 452.34 uM | Asn294, Asn205, Asp201, His279 | Leu188 | Leu188, Asp201, Asn205 |
|  |  | C11 | -4.48 | 516.33 uM | Leu188, Trp296, Leu186 | Asn803, His199 | Leu188 |
|  |  | C12 | -4.26 | 748.66 uM | Asn205, Asp201 | His279, Trp296, His199 | Asp201, Asn205, His279 |
|  |  | C13 | -4.85 | 278.19 uM | Gln147, Thr196, Asn803 | - | - |
|  |  | C14 | -5.06 | 197.07 uM | - | Tyr93, Tyr102, Asn803, His199, Trp296, Leu186, Leu188 | Leu188 |
|  |  | C15 | -5.23 | 147.83 uM | Gln147 | Tyr93, Phe100, Leu188 | Leu188- |
|  |  | C16 | -4.17 | 870.93 uM | - | His279, Thr196, His199, Leu188, Trp296, Tyr102, Tyr93, Ala804, Pro805, Tyr145, Ile281, Pro805 | His279, Ile281, Leu188, |
|  |  | C17 | -5.85 | 51.56 uM | Thr196, Phe100, Asp201, Asn294, Gln147, Asn803 | Gln147, Leu188 | Leu188, Asp201 |
|  |  | Hydralazine | -5.57 | 82.85uM | Asn205 | Ile281, Leu188, Asp201, His279 |  |
